# Effects of interventions for self-harm in children and adolescents: a systematic review and meta-analysis

**DOI:** 10.1007/s00787-025-02859-7

**Published:** 2025-09-27

**Authors:** Björn Axel Johansson, Karin Wilbe Ramsay, Agneta Pettersson, Johan Bjureberg

**Affiliations:** 1 Department of Clinical Sciences Lund, Division of Child & Adolescent Psychiatry, Lund University, Lund, and Region Skåne, Psychiatry, Habilitation & Aid, Child & Adolescent Psychiatry, Regional Inpatient Care, Malmö, Sweden; 2 Swedish Agency for Health Technology Assessment and Assessment of Social Services, Stockholm, Sweden; 3 Department of Clinical Neuroscience, Centre for Psychiatry Research, Karolinska Institutet, & Stockholm Health Care Services, Region Stockholm, Stockholm, Sweden

**Keywords:** Self-harm, Nonsuicidal self-injury, Suicide attempt, Meta-analysis, Treatment effects, Adolescents

## Abstract

Self-harm, including nonsuicidal self-injury and suicide attempts, is common among youth, associated with elevated psychopathology, suicide risk, and increased demand for clinical services. Despite advances in understanding and treatment, few interventions have demonstrated efficacy in randomized controlled trials (RCTs), which are considered the gold standard for evaluating treatment effects by randomly assigning participants to intervention or control groups. Building on prior meta-analyses, this study conducted a meta-analysis of RCTs to evaluate the efficacy of interventions in adolescents across three outcomes: self-harm regardless of suicidal intent, suicide attempts, and nonsuicidal self-injury. Systematic searches in PsycINFO, Cochrane Library, Embase, and Medline identified RCTs evaluating interventions for self-harm in youth under 18 years who engaged in self-harm at least once in the past six months. Studies were included if 80% met these criteria. Meta-analyses were conducted using random-effects modeling, primarily to account for heterogeneity across studies, as this approach accommodates variation in effect sizes that may arise from differences in for example study populations, and interventions. Certainty of evidence was assessed using GRADE. Of 6497 screened records, 21 studies met inclusion criteria. Meta-analysis showed that across three studies, Dialectical Behavior Therapy (DBT) reduced the number of adolescents engaging in self-harm behaviors (risk difference [RD]=–0.12, 95% confidence interval: − 0.22 to − 0.02), with moderate certainty. Meta-analysis of two studies found Internet-delivered Emotion Regulation Individual Therapy for Adolescents (IERITA) reduced both the episodes (mean difference =–4.65, − 8.04 to − 1.25) and occurrence of nonsuicidal self-injury at treatment end (RD=–0.20, − 0.34 to − 0.07), with low certainty of evidence. DBT-A appears effective in preventing repeated self-harm, supported by findings across multiple research groups. The less resource-intensive therapy IERITA may be beneficial for adolescents with nonsuicidal self-injury. Larger sample sizes and consensus on definitions and measurement approaches will benefit future research and clinical practice.

**Prospero registration**: ID CRD42023480178.

**Supplementary Information:**

The online version contains supplementary material available at 10.1007/s00787-025-02859-7.

## Introduction

Suicide is one of the leading causes of death among 10–18-year-olds worldwide [1]. Self-harm, as both a strong predictor of future suicide attempts and deaths [2] and the second leading cause of disability globally [3], represents a critical public health concern, with its prevalence likely rising over the past decade [4]. Recently, the Lancet Commission on self-harm defined it as “intentional self-poisoning or injury, regardless of purpose” [5]. This includes behaviors like medication overdoses, ingesting harmful substances, cutting, burning, or punching. While self-harm is a broad term encompassing both suicidal and nonsuicidal self-injury (NSSI), suicide attempts refer to self-inflicted behaviors carried out with any intent to die, whereas NSSI involves deliberate self-inflicted harm without suicidal intent.

The prevalence of self-harm, regardless of intent, has been estimated at 17% [6], with suicide attempts at 5% [7], and NSSI at 18% [8]. In the general population, the prevalence of NSSI, seems to peak between 15 and 17 years, and then level off in the transition to adulthood [9]. In addition to suicide, self-harm in adolescents is associated with other adverse outcomes in young adulthood, such as psychiatric comorbidity, increased use of inpatient care, and criminality [10–13], making early identification and intervention essential. Pharmacological treatment has to this date not shown any specific effect on self-harm in adolescents [14]. The psychological treatments for self-harm vary in terms of theories, delivery, duration, and scope. Treatments are often grounded in one or more theories such as cognitive behavioral therapy (CBT) and emotion regulation theory, and can be delivered in person, over the phone, online, or in a combination of settings. The interventions can range from brief interventions focusing on motivation to change [15, 16] to year-long family-focused CBT targeting negative thought patterns, dysfunctional behaviors, and social support [17]. In conclusion, interventions are complex and sometimes similar, which facilitates assessing their effects when analyzed as a group [14]. Several systematic reviews focusing on interventions for children and adolescents have been published in recent years [14, 18–22]. Dialectical Behavior Therapy (DBT), including its adolescent adaptation (DBT-A), aims to balance acceptance and change to help individuals manage intense emotions and improve relationships. It typically involves both individual therapy and group-based skills training. It has consistently shown positive effects on self-harm. However, findings for other therapies have been mixed across reviews and outcomes. Three examples of inconsistent findings include: (1) the efficacy of Mentalization-Based Therapy for Adolescents (MBT-A), a treatment aimed at improving understanding of one’s own and others’ mental states to enhance emotion regulation and relationships; (2) family therapies; and (3) a group therapy that incorporate elements from developmental psychology, psychodynamic and cognitive theories, and other frameworks. MBT-A showed positive effects on self-harm in reviews that included only one study [18, 19], but no effects were found in others [14, 20]. Family-based interventions were effective in reducing suicidal ideation in two reviews [18, 19], but not in a third [14]. Similarly, group therapy showed effectiveness for self-harm in one review [19], but not in two others [14, 18].

The variability in previous meta-analytic findings highlights the need for updated research to provide current evidence on the effectiveness of various treatments for self-harm — both suicide attempts and NSSI — in children and adolescents. Notably, four [14, 18, 19, 21] of the six reviews did not include studies published after 2020, and none of the reviews included studies published after 2022 [14, 18–22]. Given the general lack of effective interventions and the research-intensive nature of this field, it is likely that new studies have emerged that could offer updated insights into the efficacy of these therapies.

## Method

This systematic review with meta-analysis is part of a report [23] commissioned by the Swedish Ministry of Social Welfare and conducted by the Swedish Agency for Health Technology Assessment and Assessment of Social Services (SBU). The systematic review followed a pre-registered protocol in the PROSPERO database under ID CRD42023480178 and is reported according to the PRISMA guidelines [24].

### Inclusion criteria

RCTs on children and adolescents under 18 years who had engaged in self-harm behaviors (regardless of suicidal intent) at least once in the past six months [14], published in peer-reviewed journals and written in English were included. Studies where at least 80% of the study population fulfilled the inclusion criteria were included. This threshold was chosen to allow the inclusion of studies where a small proportion of participants fell outside the strict eligibility criteria but where the sample was still largely representative of the target population. All types of interventions (psychosocial, pharmacological, or other) were included and all types of controls (active, placebo, waitlist, or other) were accepted.

### Outcome measures

The primary outcome was self-harm regardless of intent. Secondary outcomes were suicide, suicide attempts, NSSI, suicidal ideation, depression, anxiety, and level of function. Outcomes rated by adolescents, parents, or clinicians were accepted, as well as outcomes derived from patient journals or registries. The primary follow-up time was end-of-treatment, but longer follow-up times were also investigated.

Procedures.

### Literature search

The complete search strategy is presented in electronic supplementary material (ESM) 1. In brief, we adopted the same search strategy as previously used in a Cochrane report by Witt et al. [14]. The search was based on study design (RCT) and population, without restriction to age groups, and incorporated a comprehensive list of terms related to self-harm and suicidal behavior. The databases PsycINFO (Ebsco), Cochrane Library (Wiley), Embase (Elsevier), and Medline (OvidSP) were searched in May 2023 with a final update in March 2024. The database search was complemented with a citation search in the Scopus database based on all included studies from the review by Witt et al. [14]. For identification of studies before 2020, the lists of included and excluded full-text studies in Witt et al. [14] were used and screened against our inclusion criteria.

### Screening

Screening of titles and abstracts was conducted independently by two researchers (AP, KWR) using the Covidence tool (www.covidence.org). If at least one researcher found a reference of potential interest, it was included for full-text review. Two researchers (BAJ, JB) then independently assessed each full-text article for relevance according to the inclusion criteria. Conflicts were resolved by discussion, involving the full research group when necessary. Studies that did not meet the criteria were excluded from the review. A list of excluded studies, along with the primary reason for exclusion, is available in Table S1 in ESM 2.

### Risk of bias assessment

The risk of bias was assessed separately for each outcome according to the revised Cochrane risk-of-bias tool for randomized trials (RoB 2 [25]). The risk of bias for each outcome was classified as low, some concerns, or high. The assessment was conducted by two reviewers independently (AP, BAJ, JB, KWR), and disagreements were resolved by discussion, involving the full research group when necessary. If a study was co-authored by any of the reviewers, the authoring reviewer was excluded from the risk of bias assessment and further analyses involving that study.

### Data extraction

For included studies, the following data were extracted: country of origin, study design, setting, number of study participants in total and per study arm, brief description of the study population including clinical presentation, mean age, male/female ratio, and race or ethnicity, description of the intervention and the comparison and outcome data (Table 1). Data were extracted by one reviewer and independently checked for accuracy against the original study by a second reviewer.

**Table 1:** Summary of characteristics of included clinical trials

Reference	Setting Country	Inclusion criteria for SH	Participants				Intervention			Control	Follow up time
			Mean age (Year)	Gender	Ethnicity	Co-morbidity	Therapy type	Content	Duration		
Asarnow 2017 *N* = 42	ED following SH USA	1 episode SH last 3 months Lifetime SH ≥ 3	14.6	88% F	White *N* = 35 (83,3%), Black *N* = 2 (4,8%), Hispanic/ Latino *N* = 9 (21,4%), Asian *N* = 5 (11,9%), Other *N* = 3 (7,1%)	MDD: 55%	SAFETY, family intervention (*n* = 20)	Based on CBT, DBT and with safety planning and crisis card	12 weeks	EUC (TAU + family component) *n* = 22	12 months
Bjureberg 2023 *N* = 166	Website and telephone Sweden	Diagnostic criteria for NSSID (≥ 1-episode NSSI the last months) No history of SA	15	93% F	Region of birth: Sweden *N* = 160 (96%), Asia, South or North America, or Europe *N* = 6 (4%)		IERITA + TAU	Therapist-guided, 11 modules for the adolescent and 6 for the parents *n* = 84	12 weeks	TAU (specified) *n* = 82	Up to 3 months posttest
Cotgrove 1995 *N* = 105	ED following SH UK	NR	14.9	85% F	NR	Psychiatric disorder: 6%	Self-admission (Emergency green card) *N* = 47		12 months	TAU (not specified) *N* = 58	12 months
Cottrell 2018 *N* = 832	Outpatient USA	≥ 2 SH prior to index episode	14.3	89% F	NR		Systemic family therapy (SHIFT) *n* = 415		6–8 sessions 6 months	TAU consistent with NICE guidelines *n* = 417	Up to 18 months
Dobias 2021 *N* = 565	Web Advertisement to reach LGBTQ-groups USA	Recent engagement in NSSI	15	66% F 37.5% gender differs from sex	White: 75% Hispanic/Latinx: 21,1% African-American: 9,7% Asian: 7,3% Native American Indian or Alaska Native 5,5% Other: 4,6%		Single session web-based *n* = 286	Based on CBT	30 min	Supportive therapy, 30 min online *n* = 279	Posttest and 3 months later
Donaldson 2005 *N* = 31	ED following SA USA		15	82% F	NR	MDD: 29% SUD: 50%	Individual SBT with family component *n* = 15	Based on CBT Problem solving and affect management	Acute: 3 months Booster: 3 months	Supportive relationship (analogue TAU) *n* = 16	3 and 6 months
Duarte Velez 2022 *N* = 46	Home USA	Inpatients with active SI during the past months or a SA during the two last months	15	80% F	Latinx: 100% (Latinx-White: 35% Latinx-Black: 15% Latinx-mix: 22%)	Mood disorders: 89% AD: 70% ODD: 30% CD: 26%	SCBT-SB, for child and caretaker *n* = 24	Based on CBT	1.5–3 h/week during 6–14 weeks	TAU, home based, eclectic *n* = 22	3, 6 and 12 months post baseline
Esposito Smythers 2019 *N* = 147	Outpatient care USA	Hospitalized for SA or SI. One SA prior to index admission OR NSSI OR SUD	15	76% F	White: 85,5% Black: 2,2% Asian Pacific: 2,8% Multiracial: 12%	Mood disorders 100%	F-CBT, family-focused CBT *n* = 74	Average 27 adolescent sessions and 20 parent sessions	12 months (weekly first 6 months, biweekly 6–9 months and 1/months 9–12 months)	EUC (TAU + opportunities for contact) *n* = 73	6, 12, 18 months post randomization
Green 2011 *N* = 366	CAMHS UK	≥ 2 lifetime SH episodes in 12 months preceding trial entry	NR, range 12 to 17 years	89% F	White: 93,4% Minorities: 6,6%	MDD: 62% Behavioral disorder: 33%	Group psychotherapy *n* = 183	Based on CBT, DBT, group psychotherapy	Up to 32 sessions (mean 10.1) 6 weeks + weekly boosters as needed	TAU according to clinical judgement. Group-based interventions were excluded *n* = 183	6 and 12 months
Griffiths 2019 *N* = 53	CAMHS Scotland	SH in 6 months preceding trial entry	15.6	79% F	White, Scottish born: 68,8%	33% BPD	MBT-A *n* = 22	12 sessions	12 weeks	TAU according to protocols and guidelines *n* = 26	36 weeks
Harrington 1998 *N* = 162	Home UK	Referred to outpatient care for an episode of self-poisoning	14.5	89% F	White: 90% I, 88% C Black: 1% I, 2% C Asian: 2%	67% MDD	Family therapy (*n* = 85)	5 sessions targeting communication and problem solving		TAU, not specified (*n* = 77)	6 months 6 years
Hazell 2009 *N* = 72	Outpatient Australia	≥ 2 episodes SH in the year preceding entry (1 last 3 months); ineligible if they required more intensive treatment owing to imminent danger of self-harm	14.4	90% F	NR	MDD: 57% CD/ODD: 7% SUD: 4%	Group based therapy + TAU (*n* = 35)	Based on CBT, IPT, group psychotherapy	Six weekly sessions plus optional sessions as needed	TAU incl family sessions (*n* = 37)	12 months
Kaess 2020 *N* = 74	Outpatient Referral or self-referral Germany	≥ 5 episodes NSSI in six months; one during last months Exclusion criteria: acute intent to harm self or others that required intensive psychiatric inpatient treatment	14.9	96% F	German:90% Other European: 5% Asian: 5%	Depression and dysthymia: 69% ODD: 4% SUD: 1%	Cutting Down Programme (*n* = 37)	Less intensive than TAU (based on CBT and DBT)	8–12 sessions, once weekly for 2–4 months	CBT or psychodynamic therapies (*n* = 37)	4 and 10 months past baseline
Kennard 2018 *N* = 66	Inpatient, followed by outpatient USA	Hospitalized for SI with plan or intent, or SA	15.1	89% F	White: 77,3%	MDD: 86% AD: 58%	BI, As Safe as Possible + Smartphone app BRITE with daily assessments and supportive texts (*n* = 34)	BI delivered on the inpatient unit MI framework.	1 session, 3 h,	TAU, specified (*n* = 32)	4, 13 and 24 weeks post baseline
McCauley 2018 *N* = 173	Outpatient USA	≥ 1 lifetime SA ≥ 3 lifetime SH (1 in the 12 weeks preceding trial entry) ≥ 3 criteria for BPD High risk for suicide (SIQ-JR ≥ 24)	15	94% F	White: 56,4% Hispanic: 27,5% Afro-American: 7% Native American: 0,6% Other: 2,3%	MDD: 84% AD: 54% BPD: 53%	DBT *n* = 86	Individual, group and family components	Weekly for 6 months	TAU (specified) *n* = 87	1 year
Mehlum 2014 *N* = 77	Outpatient Norway	≥ 2 episodes SH lifetime (≥ 1 within 16 w preceding trial entry) ≥ 2 criteria BPD diagnosis OR ≥ 1 criterion for diagnosis and ≥ 2 subthreshold criteria	15.6	88% F	Norwegian: 84,9%	MDD: 60% AD: 43% BPD: 26% Eating disorder: 20% SUD: 2.6%	DBT (*n* = 39)	Individual and group family component	Weekly for 19 weeks	EUC (CBT or psychodynamic therapy) (*n* = 38)	16 w post test 1 year, 3 years
Morthorst 2022 *N* = 30	Outpatient Denmark	≥ 5 episodes NSSI during last year; ≥1 episode in last month	15	97% F	Danish: 97% Other European: 3%	Affective disorder: 27% AD: 37% Personality disorders: 23%	IERITA (*n* = 15)	See Bjureberg 2023 (Bjureberg et al., 2023)	12 weeks	TAU (incl family-based treatment, CBT, DBT) (*n* = 15)	12 weeks posttest
Ougrin 2011, 2013 *N* = 70	ED UK	Admitted to ED following SH UK	15.6	80% F	White: 52% Black: 20% Asian: 11% Mixed: 13% Other: 3%	Mood disorders: 60%	Manualised enhanced therapeutic assessment	Based on MI (*n* = 35)	1 h assessment and 30 min BI	TAU following NICE guidelines (*n* = 35)	2 years
Rossouw 2012 *N* = 80	Outpatient UK	≥ 1 episode SH within the month preceding study entry	15	85% F	White: 75% Asian: 10% Black: 5% Other: 10%	MDD: 96% BPD: 72% SUD: 71%	MBT-A (*n* = 40)		Weekly (individuals) and monthly (family) 12 months	TAU following NICE guidelines (*n* = 40)	3, 6, 9, 12 months
Santamarina Peres 2020 *N* = 35	Outpatient Spain	Repetitive SH during last year and at high risk for suicide	15.2	89% F	NR	MDD: 83% AD: 54% BIP: 14%	DBT-A (*n* = 18)		Weekly for individual and family 16 weeks	EUC with family component (specified) (*n* = 17)	16 weeks (posttest)
Wood 2001 *N* = 63	Outpatient UK	≥ 2 episodes SH during last year (one is the index episode)	14	78% F	NR	MDD: 82,5%	Developmental psychotherapy, group based (*n* = 32)	See Hazell and Green (Green et al., 2011; Hazell et al., 2009)	≥ 8 weekly sessions 6 months	TAU (not specified) (*n* = 31)	7 months

*AD* = Anxiety disorder; *BIP*: Bipolar Disorder; *BPD* = Borderline Personality Disorder; *CD*: Conduct Disorder; *ED* = Emergency Department; *ERITA* = Emotion Regulation Individual Therapy for Adolescents; *IPT*: Interpersonal Psychotherapy; *MD* = Mean Difference; *NR*: not reported; *NSSI* = Nonsuicidal Self-Injury; *ODD*: Oppositional Defiant Disorder; *SA* = Suicide Attempts; *SH* = Self Harm; *SIQ* = Suicidal Ideation Questionnaire; *SIQ-JR* = Suicidal Ideation Questionnaire, Junior Version; *SUD*: Substance use disorder; *TAU* = Treatment as Usual

**Table 2:** Summary of findings for the main outcomes at end-of-treatment or first follow-up

Intervention	Outcome	No. of participants (No. of studies)	Absolute effect (95% Confidence intervals)	Certainty of the evidence (GRADE)
CBT	Self-harm definition			
	Self-harm any	NA	-	-
	NSSI	202 (2 RCT)	RD= −0.12 (−0.25 to 0.02)	Very low
	Suicide attempts	348 (5 RCT)	RD= −0.01 (−0.10 to 0.09)	Low^a^
	Suicidal ideation	193 (3 RCT)	SMD= −0.10 (−0.38 to 0.18)	Very low
DBT-A	Self-harm definition			
	Self-harm any	283 (3 RCT)	RD= −0.12 (−0.22 to −0.02)	Moderate^b^
	NSSI	NA	-	-
	Suicide attempts	208 (2 RCT)	RD= −0.04 (−0.13 to 0.05)	Very low
	Suicidal ideation	233 (3 RCT)	MD (SIQ-JR)= −9.80 (−15.16 to −4.45)	Moderate^b^
IERITA	Self-harm definition			
	Self-harm any	NA	-	-
	NSSI	196 (2 RCT)	RD= −0.20 (−0.34 to −0.07)	Low^b^
	Suicide attempts	166 (1 RCT)	RD= −0.05 (−0.13 to 0.03)	Very low
	Suicidal ideation	NA	-	-
MBT-A	Self-harm definition			
	Self-harm any	133 (2 RCT)	RD= −0.05 (−0.40 to 0.29)	Very low
	NSSI	NA	-	-
	Suicide attempts	NA	-	-
	Suicidal ideation	NA	-	-
BI: As Safe as Possible	Self-harm definition			
	Self-harm any	NA	-	-
	NSSI	66 (1 RCT)	RD = 0.01 (−0.23 to 0.24)	Very low
	Suicide attempts	66 (1 RCT)	RD= −0.13 (−0.33 to 0.06)	Very low
	Suicidal ideation	66 (1 RCT)	RD= −0.07 (−0.30 to 0.16)	Very low
BI: Therapeutic Assessment	Self-harm definition			
	Self-harm any	70 (1 RCT)	RD= −0.06 (−0.25 to 0.14)	Very low
	NSSI	NA	-	-
	Suicide attempts	NA	-	-
	Suicidal ideation	NA	-	-
BI: SAVE	Self-harm definition			
	Self-harm any	NA	-	-
	NSSI	565 (1 RCT)	NS	Very low
	Suicide attempts	NA	-	-
	Suicidal ideation	565 (1 RCT)	NS	Very low
Brief admission by self-referral	Self-harm definition			
	Self-harm any	NA	-	-
	NSSI	NA	-	-
	Suicide attempts	105 (1 RCT)	RD= −0.06 (−0.17 to 0.05)	Very low
	Suicidal ideation	NA	-	-
Group therapy	Self-harm definition			
	Self-harm any	501 (3 RCT)	RD= −0.00 (−0.23 to 0.22)	Very low
	NSSI	NA	-	-
	Suicide attempts	NA	-	-
	Suicidal ideation	414 (2 RCT)	MD = 0.47 (−7.92 to 8.86)	Moderate^a^
Systemic family therapy	Self-harm definition			
	Self-harm any	832 (1 RCT)	RD = 0.04 (−0.02 to 0.10)	Low^a^
	NSSI	NA	-	-
	Suicide attempts	NA	-	-
	Suicidal ideation	832 (1 RCT)	OR = 0.64 (0.44 to 0.94)	Moderate^b^
Home-based family therapy	Self-harm definition			
	Self-harm any	162 (1 RCT)	RD= −0.01 (−0.12 to 0.09)	
	NSSI	NA	-	-
	Suicide attempts	NA	-	-
	Suicidal ideation	154 (1 RCT)	MD= −3.40 (−19.18 to 12.38)	Very low

^a^ The evidence suggests little to no difference in effect

^b^ The evidence suggests a reduction in outcome

*BI* = Brief Interventions; *CBT* = Cognitive Behavioral Therapy; *CI* = Confidence Interval; *DBT-A* = Dialectal Behavior Therapy for Adolescents; *IERITA* = Internet-delivered Emotion Regulation Individual Therapy; *MBT-A* = Mentalization Based Therapy for Adolescents; *MD* = Mean Difference; *NA* = Not Analyzed; *NS* = Non-Significant; *NSSI* = Nonsuicidal Self-Injury, *OR* = Odds Ratio; *RCT* = Randomized Controlled Trial; *RD* = Risk Difference, *SMD* = Standardized Mean Difference

### Synthesis

Studies were categorized into intervention groups based on the study authors’ description of the content of the interventions. Meta-analyses were performed when the included studies of an intervention group were considered sufficiently homogenous in terms of population, intervention, outcome measures, and follow-up time. Only outcomes with low or moderate risk of bias were included in the synthesis. We conducted separate meta-analyses for self-harm regardless of intention, NSSI, and suicide attempts, when sufficient data were provided in the studies. The heterogeneity was handled by using a random effect model. However, when the studies within an intervention group reported heterogenous data across these outcomes, we combined self-harm regardless of intention with NSSI in the same meta-analysis, in order to minimize loss of information.

### Meta-analyses

Meta-analyses were computed using Review Manager (RevMan) (*Review Manager (RevMan)*,* Version 5.3*, 2014). The random effects model was used consistently to account for variations in the studies, especially regarding population and intervention. Dichotomous outcomes (number of events) were calculated as risk difference (RD) and continuous outcomes as mean difference (MD), or, if different instruments were used, the standardized mean difference (SMD), with 95% confidence interval. In case there was only one study available for an outcome, the MD or RD was computed in RevMan if data were available. If only the calculated effect measure was presented in the original study, we used this measure instead. When data were insufficiently reported in studies, we used supplementary data retrieved from the systematic review by Witt et al. [14], or contacted the authors of the primary studies to obtain supplementary information.

For dichotomous data, we used all randomized participants as denominators in the meta-analyses and assumed that participants with missing data had zero events. Since the drop-out rate was generally higher in the control group than in the intervention group, this can be regarded as a conservative assumption that would likely lead to an underestimation of the effect. For continuous outcomes, we used the actual number of participants who contributed with data without imputation. Data for completed suicides was extracted for each study arm but not meta-analyzed due to the very low numbers (0 or 1 event in all intervention groups).

### Assessment of the certainty of evidence

The certainty of the evidence was assessed according to Grading of Recommendations Assessment, Development, and Evaluation (GRADE), where the certainty of the evidence is expressed as high, moderate, low, or very low [26]. Each outcome is assessed separately and downgraded due to limitations in five domains: overall risk of bias across studies, inconsistency, indirectness, imprecision, and publication bias.

We assessed the certainty that there was a difference in effect between the intervention and the control group (a non-null effect), or alternatively, the certainty that there was little to no difference in effect between the two groups (a null effect).

## Results

**Fig. 1:**
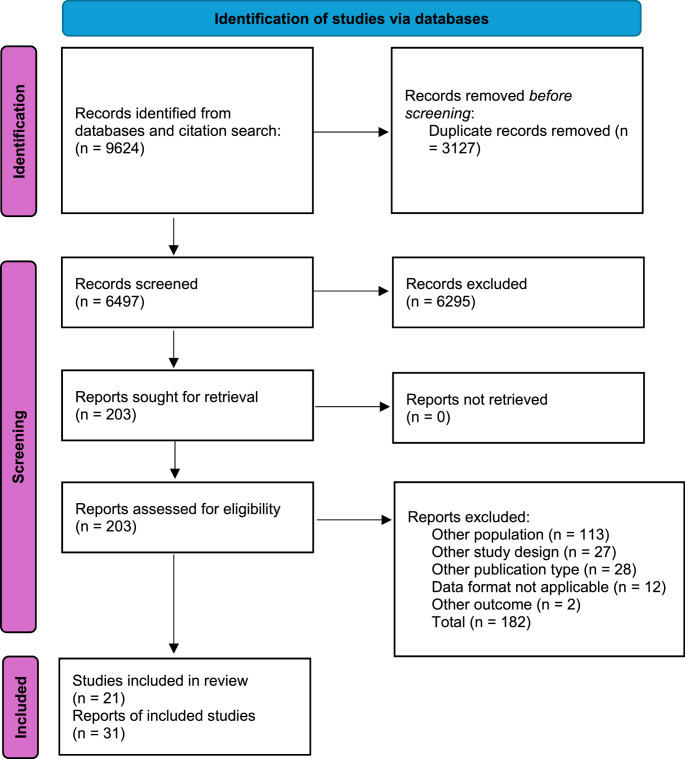
Flow diagram

See Fig. 1 for a flow chart for the selection process, conducted according to the PRISMA-guidelines [24].

### Characteristics of included studies

21 studies reported in 31 publications fulfilled our inclusion criteria and had a low risk of bias or some concerns [15–17, 27–54]. We prioritized the primary publication for each study and, where relevant, included follow-up publications if they reported additional or necessary outcome data not available in the primary report. These are summarized in Table 1. Our selection process is shown in Fig. 1 and excluded studies are summarized in Table S1 in ESM 2. All included studies were assessed as having low risk of bias or some concerns for all outcomes (See Table S2 in ESM 3).

15 studies (50%) were conducted in the USA [17, 27–29, 32–37, 41, 42, 45, 46, 55]. Seven studies (23%) were carried out in the UK [15, 16, 32, 39, 40, 51, 53]. Three studies (10%) were conducted in Norway [46–48]. One study (3%) was carried out in each of the following five countries: Spain [53], Switzerland [44], Denmark [50], Sweden [31] and Australia [43].

They included 3,263 participants aged 11 to just under 18 years, with average ages in individual studies ranging from 14.3 years [32–34] to 15.6 years [15, 16, 40, 46–48, 51]. Most participants were girls and only three studies had less than 80% girls [17, 36, 55]. Four of the studies focused on adolescents with NSSI [31, 36, 44, 50]. Thirteen studies reported data on comorbidity, mostly major depression [15, 16, 27–30, 36–38, 42–50, 52–54].

Most studies investigated psychological therapies. Brief admissions as single intervention was examined in one study [32] and as a component in a multimodal intervention in another [29]. In ten studies the intervention targeted the youth only [15, 16, 27–30, 36–38, 42–50, 52–54].

In the remaining studies the whole family participated in treatment [17, 28, 30, 32–34, 36, 37, 40, 41, 46–49, 52, 53].

The interventions usually lasted three to six months. Three studies involved short interventions of 30 min to 3 h [15, 16, 36, 45, 51] and three interventions lasted for 12 months [32, 43, 53]. TAU was the dominating choice of comparator. However, the content of TAU varied or was not specified. Four studies employed an enhanced TAU (E-TAU) adding e.g. a phone number to call in case of crisis [17, 28, 46–48, 53].

We categorized the interventions according to underlying theories to facilitate analysis of effects: Face-to face CBT, DBT-A, IERITA; MBT-A; Brief interventions; Brief admissions by self-referral; Group Therapy based on Developmental Psychotherapy and Family therapies.

### Cognitive behavioral therapy (CBT)

Five studies investigated the effects of therapies that were mainly based on CBT, delivered individually to the adolescent [37, 44], to the adolescent and the guardians separately, or to the entire family collectively [17, 29, 38]. Three studies included skills training as part of the treatment [17, 29, 38], and one study included a safety plan as a significant element of the intervention [29]. Four studies assessed the effects on repetition of suicide attempts [17, 29, 37, 38], while the fifth study focused on episodes of NSSI [44]. The duration of the treatment varied from 4 to 12 months and the number of sessions ranged from 8 to 27 (Table 1).

Meta-analyses were conducted for post-intervention and follow-up regarding SA, NSSI, suicide ideation, and depression symptoms (see Figure S1–S7 in ESM 4). Results for SA and NSSI at post-intervention are summarized in Table 2. No statistically significant differences were found between the intervention and control groups in any of the analyses (Table 2, Table S3 in ESM 4). There was little to no difference in the number of adolescents making a suicide attempt at post-intervention, RD= − 0.01 (95% CI, − 0.10 to 0.09), and at 10–12 months post-allocation, RD= −0.04 (−0.14 to 0.06), with low certainty of evidence for both timepoints. All other results were assessed as having very low certainty of evidence (Table 2, Table S3 in ESM 4).

### Dialectical behavior therapy for adolescents (DBT-A)

The effects of DBT [46] and DBT-A [46, 53] (hereafter referred to as DBT-A) were examined in three studies. For one study, follow-up data was reported in separate publications [48, 49]. The treatment duration varied from 16 to 24 weeks.

Self-harm was measured differently in the three studies; one study reported the number of adolescents with emergency department visit or hospitalization as a result of self-harm during the treatment period [46–48], another study reported on number of adolescents with any type of self-harm during the treatment period [46], and the third study reported the number of adolescents with an episode of NSSI in the past four weeks [53].

**Fig. 2:**
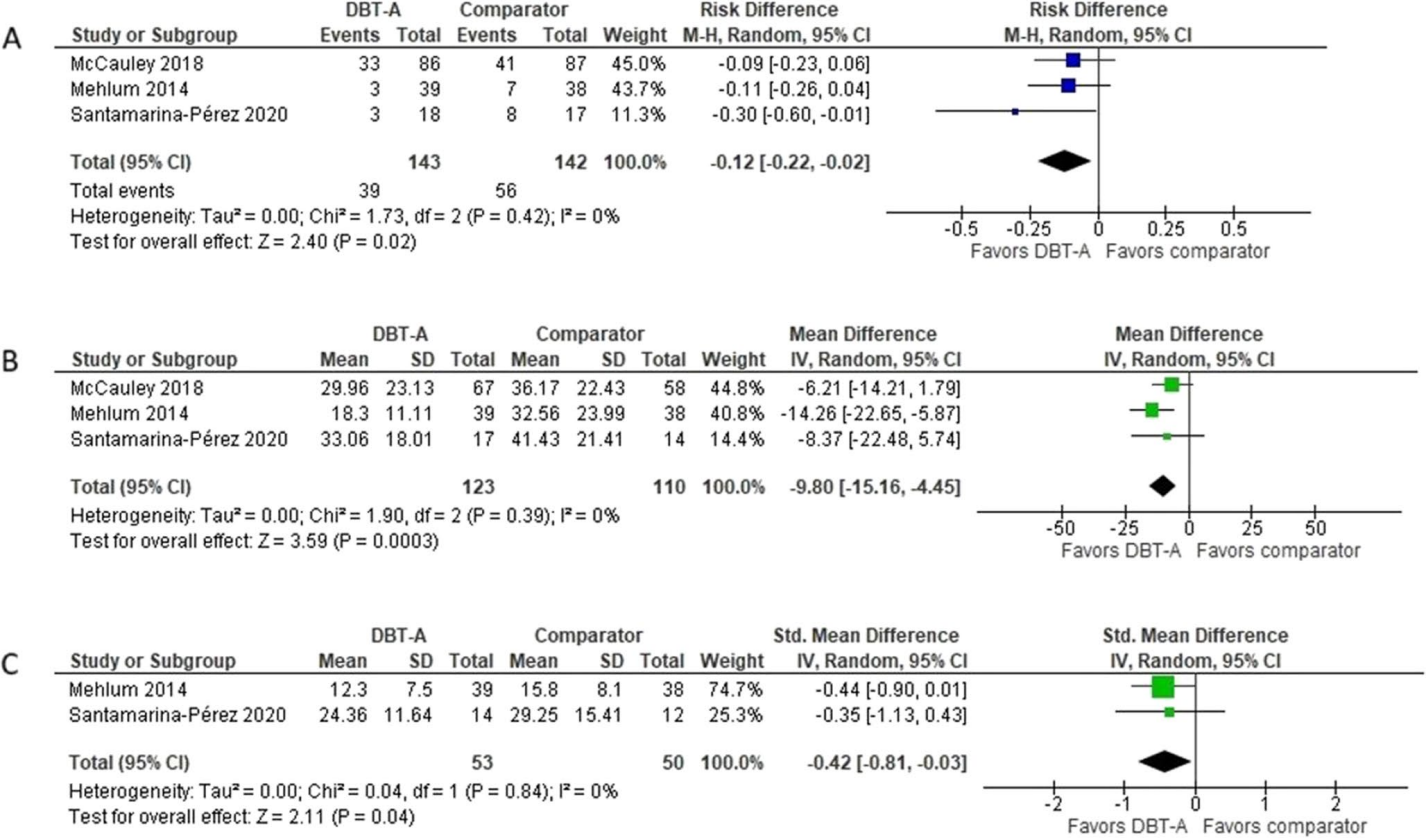
Meta-analyses of the effects of Dialectical Behavior Therapy for Adolescents (DBT-A) at post-treatment, compared to control groups, for: (**A**) self-harm, (**B**) suicidal ideation, and (**C**) depressive symptoms

Meta-analyses were conducted for post-intervention and follow-up data on SH, SA, suicide ideation, depression symptoms, and general function (see Table 2 and Figure S8–S15 in ESM 4). We found that DBT-A reduces the number of adolescents with self-harm behavior, RD= − 0.12 (95% CI, − 0.22 to − 0.02) (Table 2), and reduces suicidal ideation MD= − 9.8 (95% CI, − 15.16 to − 4.45), at post-intervention (Fig. 2). We assessed the certainty of evidence as moderate for both outcomes. In addition, we found low certainty evidence for reduced depression scores at post-intervention, SMD= −0.42 (−0.81 to −0.03). All other outcomes and follow-up analyses had very low certainty of evidence (Table S4 in ESM 4).

### Internet-delivered emotion regulation individual therapy (IERITA)

Two studies investigated the effects of therapist-supported IERITA [31, 50]. In both studies, IERITA (including asynchronous text support by therapist) in addition to TAU was compared to TAU only, which could include pharmacological treatment, support therapy, and CBT [31, 50]. The treatment lasted for 3 months in both studies. One study reported outcomes at 6 months post-allocation in addition to the end-of-treatment data [31].

**Fig. 3:**
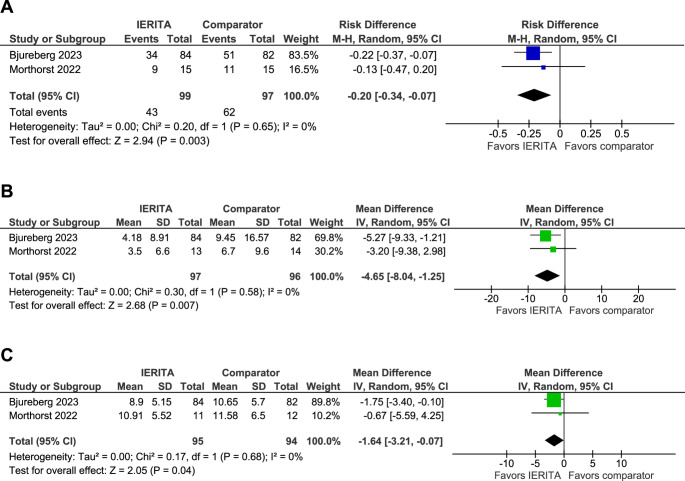
Meta-analyses of the effects of Internet-delivered Emotion Regulation Individual Therapy for Adolescence (IERITA) at post-treatment, compared to control group, for: (**A**) number of adolescents with nonsuicidal self-injury, (**B**) nonsuicidal self-injury frequency, and (**C**) depressive symptoms

Meta-analyses were conducted for the following outcomes: number of participants with NSSI, frequency of NSSI episodes, and depression and anxiety symptom scores (see Table 2 and Figure S16–S19 in ESM 4). The analyses showed that IERITA reduces the number of adolescents with NSSI at the end of treatment, RD= − 0.20 (95% CI, − 0.34 to − 0.07), as well as the frequency of NSSI episodes at the end of treatment MD= −4.65 (−8.04 to −1.25), with low certainty of evidence (Table 2, Fig. 3). We also found low certainty of evidence for a reduction of depression scores at end of treatment, MD= −1.64 (−3.21 to −0.07) (Fig. 3). Results on suicides attempts, anxiety and, general function as well as all follow-up analyses at 6 months had very low certainty of evidence (Table S5 in ESM 4).

### Mentalization-based treatment for adolescents (MBT-A)

Two studies evaluated the effects of mentalization-based therapy, MBT-A [40, 53]. One study included weekly sessions for adolescents and monthly sessions for the whole family, lasting one year [53]. In the other study, the therapy consisted of twelve weekly sessions to the adolescent alone [40].

Self-harm was self-rated in one of the studies [53]. For the other study, we used data received by the author upon request on the number of adolescents who visited an emergency department due to self-harm [40]. Meta-analyses were conducted for the following outcomes: number of participants with self-harm, and depression and anxiety symptom scores (See Table 2 and Figure S20–S24 in ESM 4). To compensate for the difference in therapy duration, analyses were performed for 3- and 8 to 9-months post-allocation data in addition to the end-of-treatment analyses. However, none of the analyses were statistically significant and we assessed the certainty of evidence as very low for all outcomes (Table S6 in ESM 4).

### Brief interventions

Brief interventions were investigated in three studies [15, 16, 36, 45, 51]. As the content of the interventions differed substantially, we did not combine their results in meta-analyses. One study evaluated As Safe As Possible (ASAP), which consisted of a three-hour session of motivational interviewing given to hospitalized participants, followed by daily self-assessment through a mobile app [45]. Another study compared enhanced psychosocial assessment (therapeutic assessment) with standard psychosocial assessment [15, 16, 51]. The enhancement consisted of a 30-minute session including motivational interviewing to encourage participation in continued treatment. The third study investigated a 30-minutes web-based, self-administered intervention based on CBT (“Project SAVE”), for adolescents with self-injurious thoughts and behaviors, which was compared to a 30-minutes web-based supportive therapy [36]. No significant results for our specified outcomes were reported from any of the studies, and we assessed all outcomes as having very low certainty of evidence (see Table S7–S8 in ESM 4).

### Brief admissions by self-referral

One study evaluated brief admissions by self-referral compared to TAU for adolescents with a previous suicide attempt [32]. The adolescents in the intervention group received an emergency green card at discharge from hospital, allowing readmission to hospital on demand. Repetition of suicide attempt was evaluated after 12 months but showed no statistically significant differences between the groups (6% in the intervention group versus 12% in the control group). We assessed the certainty of evidence as very low (Table S9 in ESM 4).

### Group therapy

Three studies, including two pilot studies [43, 55] and one larger study [39], evaluated the effectiveness of a group-based psychotherapy including components primarily from CBT, DBT-A, and psychodynamic theories. The therapy was given once a week for six to eight weeks with the possibility of additional weekly booster sessions as needed.

Meta-analyses were performed for the number of participants with self-harm (interviewed-assessed), suicide ideation scores, depression and general function scores, at 6- and 12-months post-allocation (see Table 2 and Figure S25–S32 in ESM 4). The analyses indicated that group therapy has no or a trivial effect on suicidal ideation assessed with SIQ at 6 months, MD = 0.5 (95% CI, − 7.9 to 8.9), as well as at 12 months, MD= −1.06 (−9.73 to 7.60), with moderate certainty of evidence. The analysis of self-harm also indicated a null-effect with regards to the point estimate, RD= − 0.00 (95% CI, − 0.23 to 0.22), but since the confidence interval includes both substantial positive and negative effects, we assessed the certainty of evidence as very low (Table 2 and Table S10 in ESM 4 ).

### Family therapies

Two studies evaluated interventions that were directed entirely to the family [33, 34, 41, 42]. Based on the study authors’ descriptions of the therapies, we considered them too different to combine their results, and we thus assessed them separately.

One of the studies evaluated a systematic family psychotherapy with six to eight sessions delivered over six months [33, 34]. No end-of-treatment results were reported. Follow-up assessments indicated little to no difference in the number of participants with self-harm at 12 months post-allocation, RD = 0.04 (−0.02 to 0.10), and at 3 years post-allocation: RD = 0.01 (−0.06 to 0.07). We rated the certainty of evidence as moderate regarding no or a trivial effect at both follow-up time points. A reduction of the number of adolescents with suicidal ideation was indicated at 12 months post-allocation, OR = 0.64 (95% CI, 0.44 to 0.94), but the difference was not statistically significant at 18 months post-allocation, OR = 0.76 (0.49 to 1.16). The certainty of evidence was rated as moderate for the 12 months estimate and very low for the 18 months estimate (Table S11 in ESM 4).

The intervention in the other study focused on communication and problem-solving and consisted of five sessions delivered to the family in their home [41, 42]. Results were reported for self-harm, suicide ideation and depression, but we assessed the certainty of evidence as very low for all outcomes, primarily due to the limited number of participants (Table S12 in ESM 4).

## Discussion

The results of the present meta-analysis support the efficacy of DBT in reducing self-harm and suggest that IERITA may have a potential in preventing NSSI in youth. Both DBT and IERITA appear to be potentially efficacious in reducing depression and DBT may additionally improve suicidal ideation. First-wave individual CBT possibly has no effect on suicide attempts and systemic family therapy possibly has no effect on self-harm. Group therapy and systemic family therapy may ameliorate suicidal ideation. For all other interventions and outcomes, the effects could not be assessed mainly due to few studies and few participants.

The finding that DBT-A reduces self-harm repetition compared to TAU, EUC, or alternative psychotherapies at the end of the intervention aligns with previous research [14, 18, 19]. Similarly, the lack of sufficient evidence to evaluate the effects of other interventions including pharmacological interventions on self-harm by the end of treatment is consistent with prior findings [14]. However, pharmacological interventions may be used to manage underlying psychiatric conditions contributing to self-harm but this was outside the scope of the current meta-analysis to assess. However, this is the first meta-analysis of the recently developed IERITA. DBT-A and IERITA have a common theoretical foundation, based on the theory that if youth with self-harm behaviors learn adaptive strategies to regulate their emotions and communicate their needs, their self-harm will decrease. Both treatments are part of the third wave of CBT, which incorporates first-wave CBT components but balances those change strategies with emotional awareness and acceptance. Mediation analyses have shown that the effects of DBT-A and IERITA on self-harm are mediated by improvements in emotion regulation difficulties, such as lack of emotional clarity and nonacceptance of emotional responses [28, 31, 56]. This suggests that addressing these challenges is a crucial component of treatment for reducing self-harm behaviors. DBT-A and IERITA also include extensive components aimed at parents, helping parents learn more effective ways to manage their adolescents’ emotions and behaviors, considered essential in the treatment of self-harm in youth [57, 57]. Another treatment with a strong family component is SAFETY. Although SAFETY was categorized as CBT in the current meta-analysis which did not demonstrate an effect on self-harm; the individual study with a small sample size (*N* = 42) suggested that SAFETY resulted in fewer suicide attempts compared to usual care. The therapy is specifically designed to be offered to adolescents immediately after a suicide attempt. DBT-A, IERITA, and SAFETY differ significantly in the level of time and commitment required from both care providers and families, with intensity increasing from IERITA to SAFETY and DBT-A. Importantly, these treatments were not developed to replace one another but to complement each other, as reflected in the different populations they have been studied in. IERITA has been tested for adolescents with NSSI, many of whom had frequent self-harm behavior, a history of suicide attempts, and low psychosocial functioning, though those with immediate suicide risk were excluded. In contrast, SAFETY has been successfully tested for adolescents who recently attempted suicide and are at immediate risk, but it has not shown effectiveness for NSSI. DBT-A is the most intensive treatment and has shown an effect on self-harm behaviors in adolescents at high suicide risk. These treatments may complement each other, fitting into different stages of the self-harm and suicide process. However, more randomized studies are needed to further evaluate these treatments and determine the key factors for matching specific youth and families with the interventions most likely to be beneficial.

Self-harm is a global term and covers a spectrum of different behaviors, ranging in severity and suicidal intent. Self-harm, regardless of intent, is the main outcome in this meta-analysis. This implies a weighting towards NSSI rather than suicide attempts, as NSSI is more prevalent. This should be considered when interpreting the results. Sub-group analyses on suicide attempts were conducted when this data was presented in the studies, and except for individual CBT, which showed no or negligible effects on the number of adolescents making a suicide attempt compared to usual care, we were not able to draw any conclusions, mainly due to the low number of events and studies.

This study had several limitations warranting discussion. Many of the studies included in the meta-analysis had small sample sizes, were inadequately defined and with some exceptions conducted with non-manualized TAU conditions, and the exclusion or loss to follow-up of a significant number of participants. Since most studies had relatively short follow-up periods after the intervention, the long-term effects of these interventions are still unclear. Furthermore, although the prevalence of NSSI is twice as high in girls compared to boys [8], about 90% of the participants in the included studies were girls, limiting the generalizability of the findings. It is crucial to enhance the identification of boys and non-binary individuals with self-harm behaviors in healthcare and to ensure their increased recruitment in future studies. Moreover, all included studies were conducted in Western countries, which may limit the generalizability of the findings to non-Western populations. Cultural, healthcare system, and contextual differences may influence both the implementation and effectiveness of interventions. Further, we categorized interventions based on an evaluation of their content and delivery, rather than relying solely on their labels. However, it is important to acknowledge the variation in the specific components of interventions within our aggregated results. Additionally, although individual participant data meta-analysis would allow for greater precision and deeper exploration of subgroup effects, we utilized aggregated data since this data was not readily available, which limits these capabilities. Moreover, the risk difference in the current meta-analysis was calculated using the total number of randomized participants, assuming those lost to follow-up did not engage in self-harm. This intent-to-treat approach may underestimate the effect of the experimental interventions since dropout rates were generally higher in the control group. This explains why our analysis shows a slightly smaller effect of DBT-A on self-harm compared to the Cochrane review [14], which only included participants with follow-up data.

## Conclusions

DBT-A appears to be effective in preventing repeated self-harm and is the only treatment which has shown effect in multiple studies by different research groups. IERITA may also show promise. More research and replication of positive findings by independent groups are urgently needed for IERITA and other interventions. In line with recommendations from other reviews [14, 19, 21], additional studies are crucial to identify specific interventions with proven efficacy and effectiveness in routine clinical settings, to understand the mechanisms by which interventions reduce self-harm risk, and to determine the key factors for matching specific youth and families with the interventions most likely to be beneficial. Greater international consensus on definitions and measurement strategies for self-harm behaviors will further strengthen efforts to advance research and practice.

## Supplementary Information

Below is the link to the electronic supplementary material.

ESM 1 (DOCX 43.7 KB)

ESM 2 (DOCX 57.6 KB)

ESM 3 (DOCX 347 KB)

ESM 4 (DOCX 553 KB)

## Data Availability

All data underlying the present analyses are available in the Supplemental files.
