# Supplementary material for: Effects of interventions for self-harm in children and adolescents: a systematic review and meta-analysis
Source: Eur Child Adolesc Psychiatry. 2025 Sep 27;35(1):91–107. doi: 10.1007/s00787-025-02859-7 (PMC12916994; doi:10.1007/s00787-025-02859-7)
Supplement: Supplementary file 1 — (DOCX 43.7 KB) [file 787_2025_2859_MOESM1_ESM.docx]

# Appendix 1 Search strategies

Literature search based on Witt et al. 2021^[[1]](#footnote-2)^

PsycINFO via EBSCO 4 March 2024

| Search terms | | Items found |
| --- | --- | --- |
| Population | | |
|  | DE Self-destructive Behavior | 3,464 |
|  | DE Self-inflicted Wounds | 815 |
|  | DE Suicidal Behavior | 770 |
|  | DE Suicide | 37,566 |
|  | DE Suicidal Ideation | 15,269 |
|  | DE Attempted Suicide | 12,415 |
|  | DE Self-Poisoning | 333 |
|  | DE Suicide Prevention | 6,696 |
|  | DE Suicide Prevention Centers | 142 |
|  | DE Suicidology | 475 |
|  | DE Nonsuicidal Self-Injury | 7,358 |
|  | TI ( suicid* OR parasuicid* OR "auto mutilat*" OR automutilat* OR "self destruct*" OR selfdestruct* OR self-harm* OR selfharm* OR "self immolat*" OR selfimmolat* OR "self inflict*" OR selfinflict* OR "self injur*" OR selfinjur* OR selfmutilat* OR "self mutilat*" OR self poison* OR selfpoison* OR (self N2 (cut OR cuts OR cutting OR cutter* OR burn OR burns OR burning OR bite OR bites OR biting OR hit OR hits OR hitting)) OR "head bang*" OR headbang* ) OR AB ( suicid* OR parasuicid* OR "auto mutilat*" OR automutilat* OR "self destruct*" OR selfdestruct* OR self-harm* OR selfharm* OR "self immolat*" OR selfimmolat* OR "self inflict*" OR selfinflict* OR "self injur*" OR selfinjur* OR selfmutilat* OR "self mutilat*" OR self poison* OR selfpoison* OR (self N2 (cut OR cuts OR cutting OR cutter* OR burn OR burns OR burning OR bite OR bites OR biting OR hit OR hits OR hitting)) OR "head bang*" OR headbang* ) OR KW ( suicid* OR parasuicid* OR "auto mutilat*" OR automutilat* OR "self destruct*" OR selfdestruct* OR self-harm* OR selfharm* OR "self immolat*" OR selfimmolat* OR "self inflict*" OR selfinflict* OR "self injur*" OR selfinjur* OR selfmutilat* OR "self mutilat*" OR self poison* OR selfpoison* OR (self N2 (cut OR cuts OR cutting OR cutter* OR burn OR burns OR burning OR bite OR bites OR biting OR hit OR hits OR hitting)) OR "head bang*" OR headbang* ) | 91,237 |
|  | TI ( nssi? OR ((nonsuicid* OR non-suicid*) N2 (self* OR injur*)) ) OR AB ( nssi? OR ((nonsuicid* OR non-suicid*) N2 (self* OR injur*)) ) OR KW ( nssi? OR ((nonsuicid* OR non-suicid*) N2 (self* OR injur*)) ) | 3,131 |
|  | DE Drug Overdoses | 3,031 |
|  | TX prevent* | 587,712 |
|  | S14 AND S15 | 1,143 |
|  | TI ( (nonfatal OR non-fatal) N2 (overdose* OR "over dose*") ) OR AB ( (nonfatal OR non-fatal) N2 (overdose* OR "over dose*") ) OR KW ( (nonfatal OR non-fatal) N2 (overdose* OR "over dose*") ) | 390 |
|  | *1 OR 2 OR 3 OR 4 OR 5 OR 6 OR 7 OR 8 OR 9 OR 10 OR 11 OR 12 OR 13 OR 16 OR 17* | *94,478* |
| Study types: randomised controlled trials | | |
|  | DE Randomized Controlled Trials | 1,061 |
|  | DE Randomized Clinical Trials | 522 |
|  | DE Random Sampling | 1,000 |
|  | TI ( randomi?ed OR randomi?ation OR randomi?ing ) OR AB ( randomi?ed OR randomi?ation OR randomi?ing ) OR KW ( randomi?ed OR randomi?ation OR randomi?ing ) | 113,683 |
|  | TI ( rct OR "at random" OR (random* N3 (administ* OR allocat* OR assign* OR class* OR cluster OR crossover OR cross-over OR control* OR determine* OR divide* OR division OR distribut* OR expose* OR fashion OR number* OR place* OR pragmatic OR quasi OR recruit* OR split OR subsitut* OR treat*)) ) OR AB ( rct OR "at random" OR (random* N3 (administ* OR allocat* OR assign* OR class* OR cluster OR crossover OR cross-over OR control* OR determine* OR divide* OR division OR distribut* OR expose* OR fashion OR number* OR place* OR pragmatic OR quasi OR recruit* OR split OR subsitut* OR treat*)) ) OR KW ( rct OR "at random" OR (random* N3 (administ* OR allocat* OR assign* OR class* OR cluster OR crossover OR cross-over OR control* OR determine* OR divide* OR division OR distribut* OR expose* OR fashion OR number* OR place* OR pragmatic OR quasi OR recruit* OR split OR subsitut* OR treat*)) ) | 193,595 |
|  | TI trial | 49,934 |
|  | DE Placebo | 6,627 |
|  | TI ( placebo AND (allocat* OR assign* OR control* OR group*) ) OR AB ( placebo AND (allocat* OR assign* OR control* OR group*) ) OR KW ( placebo AND (allocat* OR assign* OR control* OR group*) ) | 32,874 |
|  | AB control* N3 group* | 123,162 |
|  | TI ( control* AND (trial OR study OR group*) AND (waitlist* OR “wait* list*” OR ((treatment OR care) N2 usual)) ) OR AB ( control* AND (trial OR study OR group*) AND (waitlist* OR “wait* list*” OR ((treatment OR care) N2 usual)) ) OR KW ( control* AND (trial OR study OR group*) AND (waitlist* OR “wait* list*” OR ((treatment OR care) N2 usual)) ) | 13,751 |
|  | TI ( (single OR double OR triple OR treble) N2 (blind* OR mask* OR dummy) ) OR AB ( (single OR double OR triple OR treble) N2 (blind* OR mask* OR dummy) ) OR KW ( (single OR double OR triple OR treble) N2 (blind* OR mask* OR dummy) ) | 29,867 |
|  | DE Treatment Effectiveness Evaluation | 29,305 |
|  | *19 OR 20 OR 21 OR 22 OR 23 OR 24 OR 25 OR 26 OR 27 OR 28 OR 29 OR 30* | *366,157* |
| Combined sets | | |
|  | *18 AND 31* | *6,884* |
| Limits | | |
|  | *32 AND Publication Year: 2020-2024* | *1,819* |
| Final result | | |
|  |  | 1,819 |

The final search result, usually found at the end of the documentation, forms the list of abstracts.

**AB** = Abstract; **DE** = Term from the thesaurus; **KW** = Author keyword; **TI** = Title; ***** = Truncation; **" "** = Citation Marks; searches for an exact phrase; **?** = replaces one letter; **N** = Near Operator (N) finds the words if they are a maximum of x words apart from one another, regardless of the order in which they appear

Cochrane Library via Wiley 4 March 2024 (CENTRAL)

| Search terms | | Items found |
| --- | --- | --- |
| Population | | |
|  | MeSH descriptor: [Self-Injurious Behavior] explode all trees | 2,441 |
|  | (overdose* and prevent*):kw or (overdos* near/3 prevent*):ti,ab | 262 |
|  | ((nonfatal or non-fatal) near/2 (overdose* or over dose*)):ti,ab,kw | 64 |
|  | (NSSI* or ((nonsuicid* or non-suicid*) near/2 (self* or injur*))):ti,ab | 246 |
|  | (suicid* or parasuicid* or (auto nextmutilat*) or automutilat* or (self next destruct*) or selfdestruct* or self-harm* or selfharm* or (self next harm*) or (self next immolat*) or selfimmolat* or (self next inflict*) or selfinflict* or (self next injur*) or selfinjur* or selfmutilat* or (self next mutilat*) or (self next poison*) or selfpoison* or (self near/2 (cut or cuts or cutting or cutter* or burn or burns or burning or bite or bites or biting or hit or hits or hitting)) or (head next bang*) or headbang*):ti,ab,kw | 12,955 |
|  | *1 OR 2 OR 3 OR 4 OR 5* | *13,242* |
| Limits | | |
|  | 6 AND with Cochrane Library publication date from Jan 2020 to Mar 2024, in Trials | 4,728 |
|  | 7 AND NOT CT OR ICTRP (in EndNote) | 2,610 |
| Final result | | |
|  |  | **2,610** |

The final search result, usually found at the end of the documentation, forms the list of abstracts.

**:au** = Author; **MeSH** = Term from the Medline controlled vocabulary, including terms found below this term in the MeSH hierarchy; **:ti** = Title; **:ab** = Abstract; **:kw** = Keyword; ***** = Truncation; **" "** = Citation Marks; searches for an exact phrase; **?** = replaces one letter; **NEAR/n** = Requests terms that are within 'n' words of each other in either direction; **NEXT/n** = Requests terms that are within 'n' words of each other in the order specified

Embase via Elsevier 4 March 2024

| Search terms | | Items found |
| --- | --- | --- |
| Population | | |
|  | 'automutilation'/de | 26,544 |
|  | 'suicidal behavior'/de | 20,661 |
|  | 'self immolation'/de | 63 |
|  | 'self poisoning'/de | 2,586 |
|  | 'suicidal ideation'/de | 33,376 |
|  | 'suicide'/de | 71,564 |
|  | 'suicide attempt'/de | 41,046 |
|  | 'drug overdose'/de AND prevent*:ti,ab,kw | 4,746 |
|  | suicid*:ti,kw OR parasuicid*:ti,kw OR 'auto mutilat*':ti,kw OR automutilat*:ti,kw OR 'self destruct*':ti,kw OR selfdestruct*:ti,kw OR 'self harm*':ti,kw OR selfharm*:ti,kw OR 'self immolat*':ti,kw OR selfimmolat*:ti,kw OR 'self inflict*':ti,kw OR selfinflict*:ti,kw OR 'self injur*':ti,kw OR selfinjur*:ti,kw OR selfmutilat*:ti,kw OR 'self mutilat*':ti,kw OR 'self poison*':ti,kw OR selfpoison*:ti,kw OR ((self NEAR/2 (cut OR cuts OR cutting OR cutter? OR burn OR burns OR burning OR bite OR bites OR biting OR hit OR hits OR hitting)):ti,kw) OR 'head bang*':ti,kw OR headbang*:ti,kw | 83,928 |
|  | nssi?:ti,ab,kw OR (((nonsuicid* OR 'non-suicid*') NEAR/2 (self* OR injur*)):ti,ab,kw) | 3,217 |
|  | ((nonfatal OR 'non fatal') NEAR/2 (overdose? OR 'over dose?')):ti,ab,kw | 239 |
|  | *1 OR 2 OR 3 OR 4 OR 5 OR 6 OR 7 OR 8 OR 9 OR 10 OR 11* | *166,205* |
| Study types: randomised controlled trials | | |
|  | 'randomized controlled trial'/de | 809,970 |
|  | 'randomization'/de | 98,855 |
|  | 'controlled clinical trial'/de | 442,306 |
|  | 'controlled clinical trial'/de AND ('disease management':lnk OR 'drug therapy':lnk OR 'prevention or rehabilitation':lnk OR 'therapy':lnk) | 219,866 |
|  | 'clinical trial'/mj | 17,385 |
|  | placebo:de | 420,719 |
|  | placebo:ti,ab | 373,564 |
|  | trial:ti | 422,4064 |
|  | randomi?ed:ti,ab,kw OR randomi?ation:ti,ab,kw OR randomi?ing:ti,ab,kw | 1,215,162 |
|  | rct:ti,ab,kw OR 'at random':ti,ab,kw OR ((random* NEAR/3 (administ* OR allocat* OR assign* OR class* OR cluster OR control* OR crossover OR 'cross over' OR determine* OR divide* OR division OR distribut* OR expose* OR fashion OR number* OR place* OR pragmatic OR quasi OR recruit* OR split OR subsitut* OR treat*)):ti,ab,kw) | 1,033,017 |
|  | ((singl* OR doubl* OR trebl* OR tripl*) NEAR/3 (blind* OR mask* OR dummy)):ti,ab,kw | 293,169 |
|  | control*:ti,ab,kw AND (study:ti,ab,kw OR group*:ti,ab,kw) AND ((waitlist*:ti,ab,kw OR wait*:ti,ab,kw) AND list*:ti,ab,kw OR (((treatment OR care) NEAR/2 usual):ti,ab,kw)) | 36,782 |
|  | *13 OR 14 OR 15 OR 16 OR 17 OR 18 OR 19 OR 20 OR 21 OR 22 OR 23 OR 24* | *2,255,023* |
| Combined sets | | |
|  | *12 AND 25* | *11,162* |
| Limits | | |
|  | *26 AND (2020:py OR 2021:py OR 2022:py OR 2023:py OR 2024:py)* | *2,949* |
| Final result | | |
|  |  | 2,949 |

The final search result, usually found at the end of the documentation, forms the list of abstracts.

**/de** = Term from the EMTREE controlled vocabulary; **/exp** = Includes terms found below this term in the EMTREE hierarchy; **:ab** = Abstract; **:kw** = Author keyword ; **:ti** = Article Title; **:ti,ab** = Title or abstract; ***** = Truncation; **' '** = Citation Marks; searches for an exact phrase; **NEAR/n** = Requests terms that are within 'n' words of each other in either direction; **?** = replaces one letter

Medline via OvidSP 4 March 2024

| Search terms | | Items found |
| --- | --- | --- |
| Population | | |
|  | Self-Injurious Behavior/ | 10,246 |
|  | Self-Mutilation/ | 3,252 |
|  | Suicide/ | 41,230 |
|  | Suicidal Ideation/ | 13,692 |
|  | Suicide, Attempted/ | 23,330 |
|  | Suicide Prevention/ | 11,540 |
|  | (suicid* or parasuicid* or auto mutilat* or automutilat* or self destruct* or selfdestruct* or self-harm* or selfharm* or self immolat* or selfimmolat* or self inflict* or selfinflict* or self injur* or selfinjur* or selfmutilat* or self mutilat* or self poison* or selfpoison* or (self adj2 (cut or cuts or cutting or cutter? or burn or burns or burning or bite or bites or biting or hit or hits or hitting)) or head bang* or headbang*).ti,ab,kf. | 114,558 |
|  | (nssi? or ((nonsuicid* or non-suicid*) adj2 (self* or injur*))).ti,ab,kf. | 2,787 |
|  | Drug Overdose/ and prevent*.af. | 4,059 |
|  | ((nonfatal or non-fatal) adj2 (overdose? or over dose?)).mp. | 613 |
|  | *or/1-10* | *134,280* |
| Study types: randomised controlled trials | | |
|  | Randomized Controlled Trial/ | 609,693 |
|  | Random Allocation/ | 107,101 |
|  | Controlled Clinical Trial/ | 95,571 |
|  | Double-Blind Method/ | 177,677 |
|  | Single-Blind Method/ | 33,287 |
|  | (randomi?ed or randomi?ation or randomi?ing).ti,ab,kf. | 842,701 |
|  | (rct or "at random" or (random* adj3 (administ* or allocat* or assign* or class* or cluster or crossover or cross-over or control* or determine* or divide* or division or distribut* or expose* or fashion or number* or place* or pragmatic or quasi or recruit* or split or subsitut* or treat*))).ti,ab,kf. | 752,238 |
|  | trial.ti. | 301,346 |
|  | Placebos/ or (placebo and (allocat* or assign* or control* or group*)).ti,ab,kf. | 223,888 |
|  | (control* adj3 group*).ab. | 686,868 |
|  | (control* and (trial or study or group*) and (waitlist* or wait* list* or ((treatment or care) adj2 usual))).ti,ab,kf. | 31,259 |
|  | ((single or double or triple or treble) adj2 (blind* or mask* or dummy)).ti,ab,kf. | 203,788 |
|  | *or/12-23* | *1,971,825* |
| Combined sets | | |
|  | *11 and 24* | *7,499* |
| Limits: XX | | |
|  | *limit 25 to yr="2020 - 2024"* | *1,820* |
| Final result | | |
|  |  | 2,246 |

The final search result, usually found at the end of the documentation, forms the list of abstracts.

**.ab.** = Abstract; **exp** = Term from the Medline controlled vocabulary, including terms found below this term in the MeSH hierarchy; **.fs.** = Floating sub-heading, does not include terms found below this term in the MeSH hierarchy; **.kf.** = Author keyword; **.sh.** = Term from the Medline controlled vocabulary; **.ti.** = Title; **/** = Term from the Medline controlled vocabulary, but does not include terms found below this term in the MeSH hierarchy; ***** = Focus (if found in front of a MeSH-term); ***** = Truncation (if found at the end of a free text term); **.mp.** = Text, heading word, subject area node, title; **" "** = Citation Marks; searches for an exact phrase; **adjn** = Positional operator that lets you retrieve records that contain your terms (in any order) within a specified number (n) of words of each other; **?** = replaces one or no letters

1. Witt KG, Hetrick SE, Rajaram G, Hazell P, Taylor Salisbury TL, Townsend E, Hawton K. Interventions for self-harm in children and adolescents.Cochrane Database of Systematic Reviews 2021, Issue 3. Art. No.: CD013667.DOI: 10.1002/14651858.CD013667.pub2. [↑](#footnote-ref-2)
