# Supplementary material for: Effects of interventions for self-harm in children and adolescents: a systematic review and meta-analysis
Source: Eur Child Adolesc Psychiatry. 2025 Sep 27;35(1):91–107. doi: 10.1007/s00787-025-02859-7 (PMC12916994; doi:10.1007/s00787-025-02859-7)
Supplement: Supplementary file 2 — (DOCX 57.6 KB) [file 787_2025_2859_MOESM2_ESM.docx]

### Table S1. Studies read in full-text and excluded based on relevance

| **Reference** | **Main reason for exclusion** |
| --- | --- |
| Lithium shows no preventive benefit in veterans surviving suicide attempt. The Brown University Psychopharmacology Update. 2022;33(3):1-5. Available from: <https://doi.org/10.1002/pu.30837>. | Other patient population |
| Abbar M, Demattei C, El-Hage W, Llorca PM, Samalin L, Demaricourt P, et al. Ketamine for the acute treatment of severe suicidal ideation: double blind, randomised placebo controlled trial. BMJ. 2022;376:e067194. Available from: <https://doi.org/10.1136/bmj-2021-067194>. | Other patient population |
| Abbott-Smith S, Ring N, Dougall N, Davey J. Suicide prevention: What does the evidence show for the effectiveness of safety planning for children and young people? - A systematic scoping review. J Psychiatr Ment Health Nurs. 2023;30(5):899-910. Available from: <https://doi.org/10.1111/jpm.12928>. | Other study design |
| Ahmadi N, Pynoos R, Leuchter A, Kopelowicz A. Reminder-Focused Positive Psychiatry: Suicide Prevention Among Youths With Comorbid Posttraumatic Stress Disorder and Suicidality. Am J Psychother. 2022;75(3):114-21. Available from: <https://doi.org/10.1176/appi.psychotherapy.20200061>. | Other study design |
| Ahmed GK, Elserogy YM, Elfadl GMA, Ghada Abdelsalam K, Ali MA. Antidepressant and anti-suicidal effects of ketamine in treatment-resistant depression associated with psychiatric and personality comorbidities: A double-blind randomized trial. J Affect Disord. 2023;325:127-34. Available from: <https://doi.org/10.1016/j.jad.2023.01.005>. | Other patient population |
| Aizik-Reebs A, Yuval K, Beyene Kesete Y, Lurie I, Bernstein A. Prevalence and prevention of suicidal ideation among asylum seekers in a high-risk urban post-displacement setting. Epidemiol Psychiatr Sci. 2022;31:e76. Available from: <https://doi.org/10.1017/S2045796022000579>. | Other patient population |
| Alavi A, Sharifi B, Ghanizadeh A, Dehbozorgi G. Effectiveness of cognitive-behavioral therapy in decreasing suicidal ideation and hopelessness of the adolescents with previous suicidal attempts. Iran J Pediatr. 2013;23(4):467-72. | Other study design |
| Alavi AS, Sharifi B, Ghanizadeh A, Dehbozorgi G. Cognitive-behavioral therapy in decreasing suicidal ideation and hopelessness in depressed adolescents with previous suicidal attempt. Neuropsychiatrie de l’Enfance et de l’Adolescence. 2012;60S:S172-S3. | Conference abstract |
| Andreoli A, Burnand Y, Frambati L, Manning D, Frances A. Abandonment Psychotherapy and Psychosocial Functioning Among Suicidal Patients With Borderline Personality Disorder: A 3-Year Naturalistic Follow-Up. J Pers Disord. 2021;35(1):73-83. Available from: <https://doi.org/10.1521/pedi_2019_33_423>. | Other study design |
| Argento A, Simundic A, Mettler J, Mills DJ, Heath NL. Evaluating the Effectiveness of a Brief Mindfulness Activity in University Students With Non-Suicidal Self-Injury Engagement. Arch Suicide Res. 2022;26(2):871-85. Available from: <https://doi.org/10.1080/13811118.2020.1841052>. | Other patient population |
| Arvilommi P, Valkonen J, Lindholm L, Gaily-Luoma S, Suominen K, Gysin-Maillart A, et al. ASSIP vs. Crisis Counseling for Preventing Suicide Re-attempts: Outcome Predictor Analysis of a Randomized Clinical Trial Data. Arch Suicide Res. 2024;28(1):184-99. Available from: <https://doi.org/10.1080/13811118.2022.2151957>. | Other patient population |
| Arvilommi P, Valkonen J, Lindholm LH, Gaily-Luoma S, Suominen K, Ruishalme OM, et al. A Randomized Clinical Trial of Attempted Suicide Short Intervention Program versus Crisis Counseling in Preventing Repeat Suicide Attempts: A Two-Year Follow-Up Study. Psychother Psychosom. 2022;91(3):190-9. Available from: <https://doi.org/10.1159/000521072>. | Other patient population |
| Asarnow JR, Berk M, Hughes JL, Anderson NL. The SAFETY Program: a treatment-development trial of a cognitive-behavioral family treatment for adolescent suicide attempters. J Clin Child Adolesc Psychol. 2015;44(1):194-203. Available from: <https://doi.org/10.1080/15374416.2014.940624>. | Other study design |
| Asarnow JR, Clarke GN, Miranda JM, Edelmann AC, Sheppler CR, Firemark AJ, et al. Zero Suicide Quality Improvement: Developmental and Pandemic-Related Patterns in Youth at Risk for Suicide Attempts. Evid Based Pract Child Adolesc Ment Health. 2023;9(1):1-14. Available from: <https://doi.org/10.1080/23794925.2023.2208382>. | Other patient population |
| Asarnow JR, Porta G, Spirito A, Emslie G, Clarke G, Wagner KD, et al. 'Suicide attempts and nonsuicidal self-injury in the treatment of resistant depression in adolescents: Findings from the TORDIA study': Correction. J Am Acad Child Adolesc Psychiatry. 2020;59(3):460-. Available from: <https://doi.org/10.1016/j.jaac.2011.04.003>. | Other patient population |
| Ataie S, Gharraee B, Shahboulaghi FM. The Effectiveness of Emotion Regulation Therapy for Adolescent Females With Nonsuicidal Self-Injury Disorder: A Mixed-Method Study. J Nerv Ment Dis. 2022;210(6):411-7. Available from: <https://doi.org/10.1097/NMD.0000000000001476>. | Other study design |
| Babeva KN, Klomhaus AM, Sugar CA, Fitzpatrick O, Asarnow JR. Adolescent Suicide Attempt Prevention: Predictors of Response to a Cognitive-Behavioral Family and Youth Centered Intervention. Suicide Life Threat Behav. 2020;50(1):56-71. Available from: <https://doi.org/10.1111/sltb.12573>. | Other study design |
| Bailey B, Early T, Swartz H. Poster Session II. Bipolar Disord. 2020;22(S1):93-124. Available from: <https://doi.org/10.1111/bdi.12939>. | Conference abstract |
| Bailey E, Muhlmann C, Rice S, Nedeljkovic M, Alvarez-Jimenez M, Sander L, et al. Ethical issues and practical barriers in internet-based suicide prevention research: a review and investigator survey. BMC Med Ethics. 2020;21(1):37. Available from: <https://doi.org/10.1186/s12910-020-00479-1>. | Other patient population |
| Bailey E, Robinson J, Witt K. Interventions for youth suicide and suicide-related behaviour: An update to a systematic review. Australas Psychiatry. 2023;31(4):505-23. Available from: <https://doi.org/10.1177/10398562231187972>. | Other publication type |
| Balasa R, Lightfoot S, Cleverley K, Stremler R, Szatmari P, Alidina Z, Korczak D. Effectiveness of emergency department-based and initiated youth suicide prevention interventions: A systematic review. PLoS One. 2023;18(12):e0289035. Available from: <https://doi.org/10.1371/journal.pone.0289035>. | Other publication type |
| Batterham PJ, Christensen H, Calear AL, Werner-Seidler A, Kazan D. Rates and Predictors of Deterioration in a Trial of Internet-Delivered Cognitive Behavioral Therapy for Reducing Suicidal Thoughts. Arch Suicide Res. 2022;26(2):937-47. Available from: <https://doi.org/10.1080/13811118.2020.1848671>. | Other patient population |
| Batterham PJ, Han J, Mackinnon AJ, Werner-Seidler A, Calear AL, Wong Q, et al. Factors associated with engagement in online self-help programs among people with suicidal thoughts. J Affect Disord. 2020;265:402-9. Available from: <https://doi.org/10.1016/j.jad.2020.01.044>. | Other patient population |
| Bendit N. RANZCP Abstracts. Aust N Z J Psychiatry. 2022;56(1_suppl):3-255. Available from: <https://doi.org/10.1177/00048674221088686>. | Conference abstract |
| Betz ME, Knoepke CE, Simpson S, Siry BJ, Clement A, Saunders T, et al. An Interactive Web-Based Lethal Means Safety Decision Aid for Suicidal Adults (Lock to Live): Pilot Randomized Controlled Trial. J Med Internet Res. 2020;22(1):e16253. Available from: <https://doi.org/10.2196/16253>. | Other patient population |
| Boudreaux ED, Stanley B, Green KL, Galfalvy H, Brown GK. A randomized, controlled trial of the safety planning intervention: Research design and methods. Contemp Clin Trials. 2021;103:106291. Available from: <https://doi.org/10.1016/j.cct.2021.106291>. | Other study design |
| Brent DA. 2.5 Novel Approaches to the Assessment and Treatment of Suicidal Adolescents. J Am Acad Child Adolesc Psychiatry. 2020;59(10):S125-S6. Available from: <https://doi.org/10.1016/j.jaac.2020.07.487>. | Other publication type |
| Brent DA, Kennard BD. Impact of a brief inpatient intervention and suicide safety planning app to decrease suicidal behavior after hospital discharge. J Am Acad Child Adolesc Psychiatry. 2018;57(10):S33. Available from: <https://doi.org/10.1016/j.jaac.2018.07.142>. | Conference abstract |
| Britton PC, Conner KR, Chapman BP, Maisto SA. Motivational Interviewing to Address Suicidal Ideation: A Randomized Controlled Trial in Veterans. Suicide Life Threat Behav. 2020;50(1):233-48. Available from: <https://doi.org/10.1111/sltb.12581>. | Other patient population |
| Brown LA, Belli G, Suzuki N, Capaldi S, Foa EB. Reduction in Suicidal Ideation from Prolonged Exposure Therapy for Adolescents. J Clin Child Adolesc Psychol. 2020;49(5):651-9. Available from: <https://doi.org/10.1080/15374416.2019.1614003>. | Data format not appropriate |
| Cantor N, Kingsbury M, Warner E, Landry H, Clayborne Z, Islam R, Colman I. Young Adult Outcomes Associated With Adolescent Suicidality: A Meta-analysis. Pediatrics. 2023;151(3):1-14. Available from: <https://doi.org/10.1542/peds.2022-058113>. | Other study design |
| Canuso CM, Ionescu DF, Li X, Qiu X, Lane R, Turkoz I, et al. Esketamine Nasal Spray for the Rapid Reduction of Depressive Symptoms in Major Depressive Disorder With Acute Suicidal Ideation or Behavior. J Clin Psychopharmacol. 2021;41(5):516-24. Available from: <https://doi.org/10.1097/JCP.0000000000001465>. | Other patient population |
| Carlyle D, Green R, Inder M, Porter R, Crowe M, Mulder R, Frampton C. A Randomized-Controlled Trial of Mentalization-Based Treatment Compared With Structured Case Management for Borderline Personality Disorder in a Mainstream Public Health Service. Front Psychiatry. 2020;11:561916. Available from: <https://doi.org/10.3389/fpsyt.2020.561916>. | Other patient population |
| Chesin MS, Dave CV, Myers C, Stanley B, Kline A, Monahan M, et al. Using Mindfulness-Based Cognitive Therapy to Prevent Suicide Among High Suicide–Risk Patients Who Also Misuse Opioids: a Preliminary Probe of Feasibility and Effectiveness. Int J Ment Health Addict. 2022;21(6):3721-34. Available from: <https://doi.org/10.1007/s11469-022-00817-x>. | Other patient population |
| Chesin MS, Keilp JG, Kline A, Stanley B, Myers C, Latorre M, et al. Attentional control may be modifiable with Mindfulness-Based Cognitive Therapy to Prevent Suicide. Behav Res Ther. 2021;147:103988. Available from: <https://doi.org/10.1016/j.brat.2021.103988>. | Other patient population |
| Christensen J, Oh J, Linder K, Imhof RL, Croarkin PE, Bostwick JM, McKean A. 2.69 Reducing Suicide Risk in Transgender and Gender-Diverse Youth: A Systematic Review. J Am Acad Child Adolesc Psychiatry. 2023;62(10):S203. Available from: <https://doi.org/10.1016/j.jaac.2023.09.156>. | Other publication type |
| Cohen K, Dobias M, Morris R, Schleider J. Improving uptake of mental health crisis resources: Randomized test of a single-session intervention embedded in social media. J Behav Cogn Ther. 2023;33(1):24-34. Available from: <https://doi.org/10.1016/j.jbct.2022.12.001>. | Other patient population |
| Comtois KA, Hendricks KE, DeCou CR, Chalker SA, Kerbrat AH, Crumlish J, et al. Reducing short term suicide risk after hospitalization: A randomized controlled trial of the Collaborative Assessment and Management of Suicidality. J Affect Disord. 2023;320:656-66. Available from: <https://doi.org/10.1016/j.jad.2022.09.042>. | Other patient population |
| Conner KR, Kearns JC, Esposito EC, Pizzarello E, Wiegand TJ, Britton PC, et al. Pilot RCT of the Attempted Suicide Short Intervention Program (ASSIP) adapted for rapid delivery during hospitalization to adult suicide attempt patients with substance use problems. Gen Hosp Psychiatry. 2021;72:66-72. Available from: <https://doi.org/10.1016/j.genhosppsych.2021.07.002>. | Other patient population |
| Cooney E. Feasibility of Evaluating DBT for self-harming adolescents : a small randomised controlled trial. Auckland, N.Z.: Te Pou o Te Whakaaro Nui =The National Centre of Mental Health Research and Workforce Development Auckland, N.Z.; 2010. | Other publication type |
| Cornelis J, Barakat A, Blankers M, Peen J, Lommerse N, Eikelenboom M, et al. The effectiveness of intensive home treatment as a substitute for hospital admission in acute psychiatric crisis resolution in the Netherlands: a two-centre Zelen double-consent randomised controlled trial. Lancet Psychiatry. 2022;9(8):625-35. Available from: <https://doi.org/10.1016/S2215-0366(22)00187-0>. | Other patient population |
| Czyz EK, King CA, Biermann BJ. Motivational Interviewing-Enhanced Safety Planning for Adolescents at High Suicide Risk: A Pilot Randomized Controlled Trial. J Clin Child Adolesc Psychol. 2019;48(2):250-62. Available from: <https://doi.org/10.1080/15374416.2018.1496442>. | Data format not appropriate |
| Czyz EK, King CA, Prouty D, Micol VJ, Walton M, Nahum-Shani I. Adaptive intervention for prevention of adolescent suicidal behavior after hospitalization: a pilot sequential multiple assignment randomized trial. J Child Psychol Psychiatry. 2021;62(8):1019-31. Available from: <https://doi.org/10.1111/jcpp.13383>. | Data format not appropriate |
| Darvishi N, Farhadi M, Azmi-Naei B, Poorolajal J. The role of problem-solving skills in the prevention of suicidal behaviors: A systematic review and meta-analysis. PLoS One. 2023;18(10):e0293620. Available from: <https://doi.org/10.1371/journal.pone.0293620>. | Other study design |
| De Jaegere E, Stas P, van Heeringen K, Dumon E, van Landschoot R, Portzky G. Future-Oriented Group Training for suicidal individuals: A randomized controlled trial. Suicide Life Threat Behav. 2023;53(2):270-81. Available from: <https://doi.org/10.1111/sltb.12944>. | Other patient population |
| de Leon V, Kumar A, Nagele P, Palanca BJ, Gott B, Janski A, et al. 285. Nitrous Oxide Reduces Suicidal Ideation in Treatment-Resistant Major Depression. Biol Psychiatry. 2023;93(9):S208. Available from: <https://doi.org/10.1016/j.biopsych.2023.02.525>. | Conference abstract |
| Depp CA, Parrish EM, Chalker SA, Ehret BC, Kamarsu S, Perivoliotis D, Granholm E. Pilot feasibility trial of a brief mobile-augmented suicide prevention intervention for serious mental illness. Psychiatr Rehabil J. 2023;46(1):74-82. Available from: <https://doi.org/10.1037/prj0000547>. | Other patient population |
| Di Simplicio M, Appiah-Kusi E, Wilkinson P, Watson P, Meiser-Stedman C, Kavanagh DJ, Holmes EA. Imaginator: A Proof-of-Concept Feasibility Trial of a Brief Imagery-Based Psychological Intervention for Young People Who Self-Harm. Suicide Life Threat Behav. 2020;50(3):724-40. Available from: <https://doi.org/10.1111/sltb.12620>. | Other patient population |
| Diamond GS, Levy S. Attachment-oriented family therapy as an outpatient follow-up care for adolescents after attempted suicide. Psychotherapie im Dialog. 2012;13:41-5. | Other study design |
| Dimeff LA, Jobes DA, Koerner K, Kako N, Jerome T, Kelley-Brimer A, et al. Using a Tablet-Based App to Deliver Evidence-Based Practices for Suicidal Patients in the Emergency Department: Pilot Randomized Controlled Trial. JMIR Ment Health. 2021;8(3):e23022. Available from: <https://doi.org/10.2196/23022>. | Other patient population |
| Dimidjian S, Kaufman J, Coleman N, Levy J, Beck A, Gallop R, Segal ZV. Impact of online Mindfulness-Based Cognitive Therapy on suicidal ideation: A secondary analysis of a randomized trial of Mindful Mood Balance. J Affect Disord. 2022;301:472-7. Available from: <https://doi.org/10.1016/j.jad.2021.12.051>. | Other patient population |
| Domany Y, McCullumsmith CB. Single, Fixed-Dose Intranasal Ketamine for Alleviation of Acute Suicidal Ideation. An Emergency Department, Trans-Diagnostic Approach: A Randomized, Double-Blind, Placebo-Controlled, Proof-of-Concept Trial. Arch Suicide Res. 2022;26(3):1250-65. Available from: <https://doi.org/10.1080/13811118.2021.1878078>. | Other patient population |
| Domany Y, Shelton RC, McCullumsmith CB. Ketamine for acute suicidal ideation. An emergency department intervention: A randomized, double-blind, placebo-controlled, proof-of-concept trial. Depress Anxiety. 2020;37(3):224-33. Available from: <https://doi.org/10.1002/da.22975>. | Other patient population |
| Drabu S, Sündermann O, Hong RY. A one-week online self-compassion training reduces self-criticism and pain endurance in adults with non-suicidal self-injury ideation: A randomized-waitlist controlled study. Mindfulness. 2022;13(5):1232-45. Available from: <https://doi.org/10.1007/s12671-022-01870-2>. | Other patient population |
| Duan S, Wang H, Wilson A, Qiu J, Chen G, He Y, et al. Developing a Text Messaging Intervention to Reduce Deliberate Self-Harm in Chinese Adolescents: Qualitative Study. JMIR Mhealth Uhealth. 2020;8(6):e16963. Available from: <https://doi.org/10.2196/16963>. | Other study design |
| Feeney A, Hock RS, Freeman MP, Flynn M, Hoeppner B, Iosifescu DV, et al. The effect of single administration of intravenous ketamine augmentation on suicidal ideation in treatment-resistant unipolar depression: Results from a randomized double-blind study. Eur Neuropsychopharmacol. 2021;49:122-32. Available from: <https://doi.org/10.1016/j.euroneuro.2021.04.024>. | Other patient population |
| Fischer G, Brunner R, Parzer P, Resch F, Kaess M. Short-term psychotherapeutic treatment in adolescents engaging in non-suicidal self-injury: a randomized controlled trial. Trials. 2013;14(1):294. Available from: <https://doi.org/10.1186/1745-6215-14-294>. | Other study design |
| Franz PJ, Mou D, Kessler DT, Stubbing J, Jaroszewski AC, Ray S, et al. Digital bibliotherapy as a scalable intervention for suicidal thoughts: A randomized controlled trial. J Consult Clin Psychol. 2022;90(8):626-37. Available from: <https://doi.org/10.1037/ccp000075210.1037/ccp0000752.supp> (Supplemental). | Other patient population |
| Frey JJ, Osteen PJ, Sharpe TL, Mosby AO, Joiner T, Ahmedani B, et al. Effectiveness of man therapy to reduce suicidal ideation and depression among working-age men: A randomized controlled trial. Suicide Life Threat Behav. 2023;53(1):137-53. Available from: <https://doi.org/10.1111/sltb.12932>. | Other patient population |
| Fu DJ, Ionescu DF, Li X, Lane R, Lim P, Sanacora G, et al. Esketamine Nasal Spray for Rapid Reduction of Major Depressive Disorder Symptoms in Patients Who Have Active Suicidal Ideation With Intent: Double-Blind, Randomized Study (ASPIRE I). J Clin Psychiatry. 2020;81(3). Available from: <https://doi.org/10.4088/JCP.19m13191>. | Other patient population |
| Gabilondo A, Aristegi E, Gonzalez-Pinto A, Martin Zurimendi J, Mateos Del Pino M, Roca R, et al. Prevention of Suicidal Behavior with Telemedicine in Patients with a Recent Suicide Attempt: Is a 6-month Intervention Long Enough? Suicide Life Threat Behav. 2020;50(1):211-9. Available from: <https://doi.org/10.1111/sltb.12576>. | Other patient population |
| Gaither R, Ranney M, Peachey A, Burock J, Rogers J, Bucci L, Beaudoin FL. A feasibility study of low-dose ketamine for acute management of suicidal ideation. J Am Coll Emerg Physicians Open. 2022;3(4):e12790. Available from: <https://doi.org/10.1002/emp2.12790>. | Other study design |
| Ghahramanlou-Holloway M, LaCroix JM, Perera KU, Neely L, Grammer G, Weaver J, et al. Inpatient psychiatric care following a suicide-related hospitalization: A pilot trial of Post-Admission Cognitive Therapy in a military medical center. Gen Hosp Psychiatry. 2020;63:46-53. Available from: <https://doi.org/10.1016/j.genhosppsych.2018.11.006>. | Other patient population |
| Gibson M, Moreau N, Balzamo E, Crompton D. Peer Intervention following Suicide-Related Emergency Department Presentation: Evaluation of the PAUSE Pilot Program. Int J Environ Res Public Health. 2023;20(4). Available from: <https://doi.org/10.3390/ijerph20043763>. | Other patient population |
| Gillespie C, Joyce M, Flynn D, Corcoran P. Dialectical behaviour therapy for adolescents: a comparison of 16-week and 24-week programmes delivered in a public community setting. Child Adolesc Ment Health. 2019;24(3):266-73. Available from: <https://doi.org/10.1111/camh.12325>. | Other study design |
| Goldstein T, Merranko J, Rode N, Sylvester R, Sakolsky D, Diler R, et al. ISBD Oral Abstracts. Bipolar Disord. 2022;24(S1):26-34. Available from: <https://doi.org/10.1111/bdi.13233>. | Conference abstract |
| Goldstein TR, Merranko J, Rode N, Sylvester R, Hotkowski N, Fersch-Podrat R, et al. Dialectical Behavior Therapy for Adolescents With Bipolar Disorder: A Randomized Clinical Trial. JAMA Psychiatry. 2024;81(1):15-24. Available from: <https://doi.org/10.1001/jamapsychiatry.2023.3399>. | Other patient population |
| Goodman M, Brown GK, Galfalvy HC, Page Spears A, Sullivan SR, Kapil-Pair KN. Group ("Project Life Force") versus individual suicide safety planning: A randomized clinical trial. Contemporary Clinical Trials Communications. 2020;17:100520. | Protocol |
| Goodman M, Sullivan SR, Spears AP, Crasta D, Mitchell EL, Stanley B, et al. A Pilot Randomized Control Trial of a Dyadic Safety Planning Intervention: Safe Actions for Families to Encourage Recovery (SAFER). Couple Family Psychol. 2022;11(1):42-59. Available from: <https://doi.org/10.1037/cfp0000206>. | Other patient population |
| Greenfield B, Larson C, Hechtman L, Rousseau C, Platt R. A rapid-response outpatient model for reducing hospitalization rates among suicidal adolescents. Psychiatr Serv. 2002;53(12):1574-9. Available from: <https://doi.org/10.1176/appi.ps.53.12.1574>. | Other patient population |
| Griffiths H, Duffy F, Duffy L, Brown S, Hockaday H, Eliasson E, et al. Correction to: Efficacy of Mentalization-based group therapy for adolescents: the results of a pilot randomised controlled trial. BMC Psychiatry. 2021;21(1):142. Available from: <https://doi.org/10.1186/s12888-021-03102-8>. | Other publication type |
| Grupp-Phelan J, Stevens J, Boyd S, Cohen DM, Ammerman RT, Liddy-Hicks S, et al. Effect of a Motivational Interviewing-Based Intervention on Initiation of Mental Health Treatment and Mental Health After an Emergency Department Visit Among Suicidal Adolescents: A Randomized Clinical Trial. JAMA Netw Open. 2019;2(12):e1917941. Available from: <https://doi.org/10.1001/jamanetworkopen.2019.17941>. | Other patient population |
| Gutierrez PM, Johnson L, Podlogar MC, Hagman S, Muehler TA, Hanson J, et al. Pilot study of the Collaborative Assessment and Management of Suicidality-Group. Suicide Life Threat Behav. 2022;52(2):244-55. Available from: <https://doi.org/10.1111/sltb.12817>. | Other patient population |
| Gysin-Maillart A, Soravia L, Schwab S. Attempted suicide short intervention program influences coping among patients with a history of attempted suicide. J Affect Disord. 2020;264:393-9. Available from: <https://doi.org/10.1016/j.jad.2019.11.059>. | Other patient population |
| Gysin-Maillart AC, Jansen R, Walther S, Jobes DA, Brodbeck J, Marmet S. Longitudinal Development of Reasons for Living and Dying With Suicide Attempters: A 2-Year Follow-Up Study. Front Psychiatry. 2022;13:865831. Available from: <https://doi.org/10.3389/fpsyt.2022.865831>. | Other patient population |
| Herrmann TS, Nazarenko E, Marchand W, Day A, Merrill J, Neil M, et al. Randomized Controlled Trial of a Brief Mindfulness-Based Intervention for Suicidal Ideation Among Veterans. Mil Med. 2024;189(3-4):732-41. Available from: <https://doi.org/10.1093/milmed/usac291>. | Other patient population |
| Hill RM, Dodd CG, Gomez M, Do C, Kaplow JB. The Safety Planning Assistant: Feasibility and Acceptability of a Web-Based Suicide Safety Planning Tool for At-Risk Adolescents and Their Parents. Evid Based Pract Child Adolesc Ment Health. 2020;5(2):164-72. Available from: <https://doi.org/10.1080/23794925.2020.1759469>. | Other study design |
| Hurtado-Santiago S, Guzman-Parra J, Mayoral F, Bersabe RM. Iconic Therapy for the reduction of borderline personality disorder symptoms among suicidal youth: a preliminary study. BMC Psychiatry. 2022;22(1):224. Available from: <https://doi.org/10.1186/s12888-022-03862-x>. | Other patient population |
| Ibrahim M, Levy S, Gallop B, Krauthamer Ewing S, Hogue A, Chou J, Diamond G. Therapist Adherence to Two Treatments for Adolescent Suicide Risk: Association to Outcomes and Role of Therapeutic Alliance. Fam Process. 2022;61(1):183-97. Available from: <https://doi.org/10.1111/famp.12660>. | Data format not appropriate |
| Ilgen MA, Price AM, Coughlin LN, Pfeiffer PN, Stewart HJ, Pope E, Britton PC. Encouraging the use of the Veterans Crisis Line among high-risk Veterans: A randomized trial of a Crisis Line Facilitation intervention. J Psychiatr Res. 2022;154:159-66. Available from: <https://doi.org/10.1016/j.jpsychires.2022.07.047>. | Other patient population |
| Interian A, Chesin MS, Stanley B, Latorre M, St Hill LM, Miller RB, et al. Mindfulness-Based Cognitive Therapy for Preventing Suicide in Military Veterans: A Randomized Clinical Trial. J Clin Psychiatry. 2021;82(5). Available from: <https://doi.org/10.4088/JCP.20m13791>. | Other patient population |
| Inui-Yukawa M, Miyaoka H, Yamamoto K, Kamijo Y, Takai M, Yonemoto N, et al. Effectiveness of assertive case management for patients with suicidal intent. Psychiatry Res. 2021;304:114125. Available from: <https://doi.org/10.1016/j.psychres.2021.114125>. | Other patient population |
| Ionescu DF, Fu DJ, Qiu X, Lane R, Lim P, Kasper S, et al. Esketamine Nasal Spray for Rapid Reduction of Depressive Symptoms in Patients With Major Depressive Disorder Who Have Active Suicide Ideation With Intent: Results of a Phase 3, Double-Blind, Randomized Study (ASPIRE II). Int J Neuropsychopharmacol. 2021;24(1):22-31. Available from: <https://doi.org/10.1093/ijnp/pyaa068>. | Other patient population |
| Jerant A, Duberstein P, Kravitz RL, Stone DM, Cipri C, Franks P. Tailored Activation of Middle-Aged Men to Promote Discussion of Recent Active Suicide Thoughts: a Randomized Controlled Trial. J Gen Intern Med. 2020;35(7):2050-8. Available from: <https://doi.org/10.1007/s11606-020-05769-3>. | Other patient population |
| Kalmbach DA, Cheng P, Ahmedani BK, Peterson EL, Reffi AN, Sagong C, et al. Cognitive-behavioral therapy for insomnia prevents and alleviates suicidal ideation: insomnia remission is a suicidolytic mechanism. Sleep. 2022;45(12). Available from: <https://doi.org/10.1093/sleep/zsac251>. | Other patient population |
| Kalmbach DA, Cheng P, Ahmedani BK, Peterson EL, Reffi AN, Sagong C, et al. Cognitive-behavioral therapy for insomnia prevents and alleviates suicidal ideation: insomnia remission is a suicidolytic mechanism. Sleep. 2022;45(12):A299. Available from: <https://doi.org/10.1093/sleep/zsac251>. | Conference abstract |
| Katsivarda C, Assimakopoulos K, Jelastopulu E. Communication-based suicide prevention after the first attempt. A systematic review. Psychiatriki. 2021;32(1):51-8. Available from: <https://doi.org/10.22365/jpsych.2021.003>. | Other publication type |
| Katz IR, Rogers MP, Lew R, Thwin SS, Doros G, Ahearn E, et al. Lithium Treatment in the Prevention of Repeat Suicide-Related Outcomes in Veterans With Major Depression or Bipolar Disorder: A Randomized Clinical Trial. JAMA Psychiatry. 2022;79(1):24-32. Available from: <https://doi.org/10.1001/jamapsychiatry.2021.3170>. | Other patient population |
| Keilp JG, Madden SP, Marver JE, Frawley A, Burke AK, Herzallah MM, et al. Effects of Ketamine Versus Midazolam on Neurocognition at 24 Hours in Depressed Patients With Suicidal Ideation. J Clin Psychiatry. 2021;82(6). Available from: <https://doi.org/10.4088/JCP.21m13921>. | Other patient population |
| Khazanov GK, Xu C, Hollon SD, DeRubeis RJ, Thase ME. Adding cognitive therapy to antidepressant medications decreases suicidal ideation. J Affect Disord. 2021;281:183-91. Available from: <https://doi.org/10.1016/j.jad.2020.12.032>. | Other patient population |
| Kheirabadi D, Kheirabadi GR, Mirlohi Z, Tarrahi MJ, Norbaksh A. Comparison of Rapid Antidepressant and Antisuicidal Effects of Intramuscular Ketamine, Oral Ketamine, and Electroconvulsive Therapy in Patients With Major Depressive Disorder: A Pilot Study. J Clin Psychopharmacol. 2020;40(6):588-93. Available from: <https://doi.org/10.1097/JCP.0000000000001289>. | Other patient population |
| King C, Arango A, Kramer A, Busby D, Czyz E, Foster CE. 6.35. Effect of a Youth-Nominated Support Team on 10- to 12-year mortality outcomes of suicidal adolescents: an RCT. J Am Acad Child Adolesc Psychiatry. 2018;57:s260. | Conference abstract |
| King CA, Arango A, Kramer A, Busby D, Czyz E, Foster CE, et al. Association of the Youth-Nominated Support Team Intervention for Suicidal Adolescents With 11- to 14-Year Mortality Outcomes: Secondary Analysis of a Randomized Clinical Trial. JAMA Psychiatry. 2019;76(5):492-8. Available from: <https://doi.org/10.1001/jamapsychiatry.2018.4358>. | Other patient population |
| Kodish T, Lau AS, Belin TR, Berk MS, Asarnow JR. Improving Care Linkage for Racial-Ethnic Minority Youths Receiving Emergency Department Treatment for Suicidality: SAFETY-A. Psychiatr Serv. 2023;74(4):419-22. Available from: <https://doi.org/10.1176/appi.ps.20220129>. | Wrong outcomes |
| Korczak DJ. 32.4 #IamSafe: A Multi-Site Randomized Controlled Trial of a Youth and Family-Based Suicide Prevention Intervention for Adolescents Presenting to the Emergency Department With Suicide-Risk Behaviors. J Am Acad Child Adolesc Psychiatry. 2022;61(10):S326. Available from: <https://doi.org/10.1016/j.jaac.2022.07.736>. | Conference abstract |
| Kruzan KP, Whitlock J, Bazarova NN, Bhandari A, Chapman J. Use of a Mobile Peer Support App Among Young People With Nonsuicidal Self-injury: Small-scale Randomized Controlled Trial. JMIR Form Res. 2022;6(1):e26526. Available from: <https://doi.org/10.2196/26526>. | Other patient population |
| Lee DJ, Bryan CJ, Rudd MD. Longitudinal suicide ideation trajectories in a clinical trial of brief CBT for U.S. military personnel recently discharged from psychiatric hospitalization. Psychiatry Res. 2020;293:113335. Available from: <https://doi.org/10.1016/j.psychres.2020.113335>. | Other patient population |
| Lin YC, Liu SI, Chen SC, Sun FJ, Huang HC, Huang CR, Chiu YC. Brief Cognitive-based Psychosocial Intervention and Case Management for Suicide Attempters Discharged from the Emergency Department in Taipei, Taiwan: A Randomized Controlled Study. Suicide Life Threat Behav. 2020;50(3):688-705. Available from: <https://doi.org/10.1111/sltb.12626>. | Other patient population |
| Lindkvist RM, Westling S, Eberhard S, Johansson BA, Rask O, Landgren K. 'A Safe Place Where I Am Welcome to Unwind When I Choose to'-Experiences of Brief Admission by Self-Referral for Adolescents Who Self-Harm at Risk for Suicide: A Qualitative Study. Int J Environ Res Public Health. 2021;19(1). Available from: <https://doi.org/10.3390/ijerph19010300>. | Other study design |
| LoParo D, Mack SA, Patterson B, Negi LT, Kaslow NJ. The Efficacy of Cognitively-Based Compassion Training for African American Suicide Attempters. Mindfulness. 2018;9(6):1941-54. Available from: <https://doi.org/10.1007/s12671-018-0940-1>. | Other patient population |
| Luxton DD, Smolenski DJ, Reger MA, Relova RMV, Skopp NA. Caring E-mails for Military and Veteran Suicide Prevention: A Randomized Controlled Trial. Suicide Life Threat Behav. 2020;50(1):300-14. Available from: <https://doi.org/10.1111/sltb.12589>. | Other patient population |
| Malakouti SK, Nojomi M, Ghanbari B, Rasouli N, Khaleghparast S, Farahani IG. Aftercare and Suicide Reattempt Prevention in Tehran, Iran. Crisis. 2022;43(1):18-27. Available from: <https://doi.org/10.1027/0227-5910/a000754>. | Other patient population |
| Malakouti SK, Nojomi M, Ghanbari B, Rasouli N, Khaleghparast S, Farahani IG. Aftercare and Suicide Reattempt Prevention in Tehran, Iran. Crisis. 2022;43(1):18-27. Available from: <https://doi.org/10.1027/0227-5910/a000754>. | Other patient population |
| Martin Villalba I. E-Poster Viewing. Eur Psychiatry. 2020;63(S1):S283-S589. Available from: <https://doi.org/10.1192/j.eurpsy.2020.6>. | Conference abstract |
| Masland SR, Finch EF, Schnell SE. Effects of Group Psychotherapy for Nonsuicidal Self-Injury: A Meta-Analysis. Int J Group Psychother. 2023;73(3):183-201. Available from: <https://doi.org/10.1080/00207284.2023.2191683>. | Other study design |
| McGillivray L, Keng-Meng Hui N, Wong QJJ, Han J, Qian J, Torok M. The Effectiveness of a Smartphone Intervention Targeting Suicidal Ideation in Young Adults: Randomized Controlled Trial Examining the Influence of Loneliness. JMIR Ment Health. 2023;10:e44862. Available from: <https://doi.org/10.2196/44862>. | Other patient population |
| McMain SF, Chapman AL, Kuo JR, Dixon-Gordon KL, Guimond TH, Labrish C, et al. The Effectiveness of 6 versus 12 Months of Dialectical Behavior Therapy for Borderline Personality Disorder: A Noninferiority Randomized Clinical Trial. Psychother Psychosom. 2022;91(6):382-97. Available from: <https://doi.org/10.1159/000525102>. | Other patient population |
| Mehta S, Downar J, Mulsant BH, Voineskos D, Daskalakis ZJ, Weissman CR, et al. Effect of high frequency versus theta-burst repetitive transcranial magnetic stimulation on suicidality in patients with treatment-resistant depression. Acta Psychiatr Scand. 2022;145(5):529-38. Available from: <https://doi.org/10.1111/acps.13412>. | Other patient population |
| Michel K, Gysin-Maillart A, Breit S, Walther S, Pavlidou A. Psychopharmacological treatment is not associated with reduced suicide ideation and reattempts in an observational follow-up study of suicide attempters. J Psychiatr Res. 2021;140:180-6. Available from: <https://doi.org/10.1016/j.jpsychires.2021.05.068>. | Other study design |
| Mubarak AR, Zeitz J, Slee P. Effectiveness of an intervention programme for teenage girls with self-harm in Adelaide, South Australia. Child and Adolescent Wellbeing and Violence Prevention in School. 2017:209-16. | Other publication type |
| Muhlmann C, Madsen T, Hjorthoj C, Forman JL, Kerkhof A, Nordentoft M, Erlangsen A. Effectiveness of an Internet-Based Self-help Therapy Program for Suicidal Ideation With Follow-up at 6 Months: Results of a Randomized Controlled Trial. J Clin Psychiatry. 2021;82(5). Available from: <https://doi.org/10.4088/JCP.20m13803>. | Other patient population |
| Nash AI, Turkoz I, Fu DJ, Verbanac J, Lanera L. ACNP 59(th) Annual Meeting: Poster Session III. Neuropsychopharmacology. 2020;45(Suppl 1):278-382. Available from: <https://doi.org/10.1038/s41386-020-00892-5>. | Conference abstract |
| Niederkrotenthaler T, Baumgartner J, Kautzky A, Fellinger M, Jahn R, Wippel A, et al. Effects of media stories featuring coping with suicidal crises on psychiatric patients: Randomized controlled trial. Eur Psychiatry. 2021;64(1):e70. Available from: <https://doi.org/10.1192/j.eurpsy.2021.2244>. | Other patient population |
| Niederkrotenthaler T, Till B. Effects of awareness material featuring individuals with experience of depression and suicidal thoughts on an audience with depressive symptoms: Randomized controlled trial. J Behav Ther Exp Psychiatry. 2020;66:101515. Available from: <https://doi.org/10.1016/j.jbtep.2019.101515>. | Other patient population |
| Niederkrotenthaler T, Till B. Effects of suicide awareness materials on individuals with recent suicidal ideation or attempt: online randomised controlled trial. Br J Psychiatry. 2020;217(6):693-700. Available from: <https://doi.org/10.1192/bjp.2019.259>. | Other patient population |
| Nierenberg A, Lavin P, Javitt DC, Shelton R, Matthew S, Besthof RA, Javitt JC. NRX-101 (D-cycloserine plus lurasidone) vs. lurasidone for the maintenance of initial stabilization after ketamine in patients with severe bipolar depression with acute suicidal ideation and behavior: A randomized prospective phase 2 trial. 2022. | Other patient population |
| Norimoto K, Ikeshita K, Kishimoto T, Okuchi K, Yonemoto N, Sugimoto T, et al. Effect of assertive case management intervention on suicide attempters with comorbid Axis I and II psychiatric diagnoses: secondary analysis of a randomised controlled trial. BMC Psychiatry. 2020;20(1):311. Available from: <https://doi.org/10.1186/s12888-020-02723-9>. | Other patient population |
| O'Connor RC, Smillie S, McClelland H, Lundy JM, Stewart C, Syrett S, et al. SAFETEL: a pilot randomised controlled trial to assess the feasibility and acceptability of a safety planning and telephone follow-up intervention to reduce suicidal behaviour. Pilot Feasibility Stud. 2022;8(1):156. Available from: <https://doi.org/10.1186/s40814-022-01081-5>. | Other patient population |
| O'Connor SS, McClay MM, Choudhry S, Shields AD, Carlson R, Alonso Y, et al. Pilot randomized clinical trial of the Teachable Moment Brief Intervention for hospitalized suicide attempt survivors. Gen Hosp Psychiatry. 2020;63:111-8. Available from: <https://doi.org/10.1016/j.genhosppsych.2018.08.001>. | Other patient population |
| Ohlis A, Bjureberg J, Ojala O, Kerj E, Hallek C, Fruzzetti AE, Hellner C. Experiences of dialectical behaviour therapy for adolescents: A qualitative analysis. Psychol Psychother. 2023;96(2):410-25. Available from: <https://doi.org/10.1111/papt.12447>. | Other study design |
| Ougrin D, Corrigall R, Stahl D, Poole J, Zundel T, Wait M, et al. Supported discharge service versus inpatient care evaluation (SITE): a randomised controlled trial comparing effectiveness of an intensive community care service versus inpatient treatment as usual for adolescents with severe psychiatric disorders: self-harm, functional impairment, and educational and clinical outcomes. Eur Child Adolesc Psychiatry. 2021;30(9):1427-36. Available from: <https://doi.org/10.1007/s00787-020-01617-1>. | Other patient population |
| Owens C, Fox F, Redwood S, Davies R, Foote L, Salisbury N, et al. Measuring outcomes in trials of interventions for people who self-harm: qualitative study of service users' views. BJPsych Open. 2020;6(2):e22. Available from: <https://doi.org/10.1192/bjo.2019.93>. | Other patient population |
| Owens D, Wright-Hughes A, Graham L, Blenkiron P, Burton K, Collinson M, et al. Problem-solving therapy rather than treatment as usual for adults after self-harm: a pragmatic, feasibility, randomised controlled trial (the MIDSHIPS trial). Pilot Feasibility Stud. 2020;6(1):119. Available from: <https://doi.org/10.1186/s40814-020-00668-0>. | Other patient population |
| Pachankis JE, Soulliard ZA, Layland EK, Behari K, Seager van Dyk I, Eisenstadt BE, et al. Guided LGBTQ-affirmative internet cognitive-behavioral therapy for sexual minority youth's mental health: A randomized controlled trial of a minority stress treatment approach. Behav Res Ther. 2023;169:104403. Available from: <https://doi.org/10.1016/j.brat.2023.104403>. | Other patient population |
| Pan F, Mou T, Shao J, Chen H, Tao S, Wang L, et al. Effects of neuronavigation-guided rTMS on serum BDNF, TrkB and VGF levels in depressive patients with suicidal ideation. J Affect Disord. 2023;323:617-23. Available from: <https://doi.org/10.1016/j.jad.2022.11.059>. | Other patient population |
| Pan F, Shen Z, Jiao J, Chen J, Li S, Lu J, et al. Neuronavigation-Guided rTMS for the Treatment of Depressive Patients With Suicidal Ideation: A Double-Blind, Randomized, Sham-Controlled Trial. Clin Pharmacol Ther. 2020;108(4):826-32. Available from: <https://doi.org/10.1002/cpt.1858>. | Other patient population |
| Pathak U, Ahuja SK, Dwivedi R, Mishra N, Kumar P, Mishra DK, Singh R. Antisuicidal efficacy of ketamine infusion in suicidal patients of depressive disorder. Indian J Psychiatry. 2021;63(5):483-9. Available from: <https://doi.org/10.4103/indianjpsychiatry.indianjpsychiatry_80_21>. | Other patient population |
| Pérez PS, Cela SR, Blanco IM, Martínez EF, Rossi MP, Mallén EM, Liñán AM. Efficacy of dialectical behavior therapy compared to supportive therapy in adolescents with suicidal behavior. Eur Neuropsychopharmacol. 2017;27:S853-S4. | Conference abstract |
| Petrovic J, Bastien L, Mettler J, Heath NL. The Effectiveness of a Mindfulness Induction as a Buffer Against Stress Among University Students With and Without a History of Self-Injury. Psychol Rep. 2023;126(5):2280-302. Available from: <https://doi.org/10.1177/00332941221089282>. | Other patient population |
| Phillips JL, Norris S, Talbot J, Hatchard T, Ortiz A, Birmingham M, et al. Single and repeated ketamine infusions for reduction of suicidal ideation in treatment-resistant depression. Neuropsychopharmacology. 2020;45(4):606-12. Available from: <https://doi.org/10.1038/s41386-019-0570-x>. | Other patient population |
| Pineda J, Dadds MR. Family intervention for adolescents with suicidal behavior: a randomized controlled trial and mediation analysis. J Am Acad Child Adolesc Psychiatry. 2013;52(8):851-62. Available from: <https://doi.org/10.1016/j.jaac.2013.05.015>. | Data format not appropriate |
| Pistorello J, Jobes DA, Gallop R, Compton SN, Locey NS, Au JS, et al. A Randomized Controlled Trial of the Collaborative Assessment and Management of Suicidality (CAMS) Versus Treatment as Usual (TAU) for Suicidal College Students. Arch Suicide Res. 2021;25(4):765-89. Available from: <https://doi.org/10.1080/13811118.2020.1749742>. | Other patient population |
| Pitts BH, Doyle R, Wood L, Dar R, De Jesus Ayala S, Sharma T, et al. Brief Interventions for Suicidal Youths in Medical Settings: A Meta-Analysis. Pediatrics. 2024;153(3):01. Available from: <https://doi.org/10.1542/peds.2023-061881>. | Other study design |
| Ratnaweera N, Hunt K, Camp J. A Qualitative Evaluation of Young People's, Parents' and Carers' Experiences of a National and Specialist CAMHS Dialectical Behaviour Therapy Outpatient Service. Int J Environ Res Public Health. 2021;18(11). Available from: <https://doi.org/10.3390/ijerph18115927>. | Other study design |
| Rengasamy M, Phelps-Tschang J, Simpson M, Tew JD, Jr., Sparks G. 6.50 Reduction of adolescent suicide attempts after telephone-based intervention. J Am Acad Child Adolesc Psychiatry. 2018;57. | Conference abstract |
| Rengasamy M, Sparks G. Reduction of Postdischarge Suicidal Behavior Among Adolescents Through a Telephone-Based Intervention. Psychiatr Serv. 2019;70(7):545-52. Available from: <https://doi.org/10.1176/appi.ps.201800421>. | Data format not appropriate |
| Riblet NB, Kenneally L, Stevens S, Watts BV, Gui J, Forehand J, et al. A virtual, pilot randomized trial of a brief intervention to prevent suicide in an integrated healthcare setting. Gen Hosp Psychiatry. 2022;75:68-74. Available from: <https://doi.org/10.1016/j.genhosppsych.2022.02.002>. | Other patient population |
| Riblet NB, Stevens SP, Watts BV, Gui J, Forehand J, Cornelius S, et al. A Pilot Randomized Trial of a Brief Intervention to Prevent Suicide After Inpatient Psychiatric Discharge. Psychiatr Serv. 2021;72(11):1320-3. Available from: <https://doi.org/10.1176/appi.ps.202000537>. | Other patient population |
| Roberts V, Joiner R, Russell C, Bradley R, Bowles M, Bowes A, Nice T. Mind and Body: an early intervention group programme for adolescents with self-harm thoughts and behaviours. Educ Health. 2021;37(2). | Other study design |
| Rockstroh F, Edinger A, Fischer-Waldschmidt G, Josi J, Brunner R, Resch F, et al. Sessions Abstracts. Early Interv Psychiatry. 2023;17(S1):6-203. Available from: <https://doi.org/10.1111/eip.13408>. | Conference abstract |
| Rodante DE, Kaplan MI, Olivera Fedi R, Gagliesi P, Pascali A, Jose Quintero PS, et al. CALMA, a Mobile Health Application, as an Accessory to Therapy for Reduction of Suicidal and Non-Suicidal Self-Injured Behaviors: A Pilot Cluster Randomized Controlled Trial. Arch Suicide Res. 2022;26(2):801-18. Available from: <https://doi.org/10.1080/13811118.2020.1834476>. | Other patient population |
| Rossouw T. Mentalisation based treatment for adolescents with self harm: an RCT. Eur Child Adolesc Psychiatry. 2015;24(1):S113. | Conference abstract |
| Rostami M, Azad E, Rahmati F. Effectiveness of Brief Cognitive-Behavioral Therapy on Reducing Suicide Ideation in Soldiers. J Mil Med. 2021;23(6):499-507. Available from: <https://doi.org/10.30491/JMM.23.6.499>. | Other patient population |
| Rowe SL, Patel K, French RS, Henderson C, Ougrin D, Slade M, Moran P. Web-Based Decision Aid to Assist Help-Seeking Choices for Young People Who Self-Harm: Outcomes From a Randomized Controlled Feasibility Trial. JMIR Ment Health. 2018;5(1):e10. Available from: <https://doi.org/10.2196/mental.8098>. | Data format not appropriate |
| Russon J, Abbott CH, Jin B, Rivers AS, Winston-Lindeboom P, Kobak R, Diamond GS. Attachment-based family therapy versus nondirective supportive therapy for lesbian, gay, bisexual and questioning adolescents with depression, and suicidal ideation: An exploratory study. Suicide Life Threat Behav. 2023;53(6):958-67. Available from: <https://doi.org/10.1111/sltb.12995>. | Other patient population |
| Santamarina P, Blanco IM, Picado M, Font E, Moreno E, Martínez E, Romero S. 3.65 dialectical behavior therapy versus supportive therapy for adolescents with suicidal behavior: A randomized-controlled trial. J Am Acad Child Adolesc Psychiatry. 2017;56(10):S226-S7. | Conference abstract |
| Santel M, Neuner F, Berg M, Steuwe C, Jobes DA, Driessen M, Beblo T. The Collaborative Assessment and Management of Suicidality compared to enhanced treatment as usual for inpatients who are suicidal: A randomized controlled trial. Front Psychiatry. 2023;14:1038302. Available from: <https://doi.org/10.3389/fpsyt.2023.1038302>. | Other patient population |
| Sarubbi S, Rogante E, Erbuto D, Cifrodelli M, Sarli G, Polidori L, et al. The Effectiveness of Mobile Apps for Monitoring and Management of Suicide Crisis: A Systematic Review of the Literature. J Clin Med. 2022;11(19). Available from: <https://doi.org/10.3390/jcm11195616>. | Other publication type |
| Sedghy Z, Yoosefi N, Navidian A. The effect of motivational interviewing-based training on the rate of using mental health services and intensity of suicidal ideation in individuals with suicide attempt admitted to the emergency department. J Educ Health Promot. 2020;9:247. Available from: <https://doi.org/10.4103/jehp.jehp_344_20>. | Other patient population |
| Shahzad S, Ullah S, Nazar Z, Riaz M, Khuda F, Khalil AAK, et al. Assessment of Contributing Factors and Treatment Practices for Therapeutic Efficacy and Drug-Related Problems in Suicidal Psychotic Patients. Brain Sci. 2022;12(5). Available from: <https://doi.org/10.3390/brainsci12050543>. | Other patient population |
| Simoes RMP, Dos Santos JCP, Martinho M. Adolescents with suicidal behaviours: A qualitative study about the assessment of Inpatient Service and Transition to Community. J Psychiatr Ment Health Nurs. 2021;28(4):622-31. Available from: <https://doi.org/10.1111/jpm.12707>. | Other study design |
| Simon GE, Shortreed SM, Rossom RC, Beck A, Clarke GN, Whiteside U, et al. Effect of Offering Care Management or Online Dialectical Behavior Therapy Skills Training vs Usual Care on Self-harm Among Adult Outpatients With Suicidal Ideation: A Randomized Clinical Trial. JAMA. 2022;327(7):630-8. Available from: <https://doi.org/10.1001/jama.2022.0423>. | Other patient population |
| Sinyor M, Williams M, Mitchell R, Zaheer R, Bryan CJ, Schaffer A, et al. Cognitive behavioral therapy for suicide prevention in youth admitted to hospital following an episode of self-harm: A pilot randomized controlled trial. J Affect Disord. 2020;266:686-94. Available from: <https://doi.org/10.1016/j.jad.2020.01.178>. | Data format not appropriate |
| Slep AMS, Heyman RE, Lorber MF, Baucom KJW, Linkh DJ. Evaluating the Effectiveness of NORTH STAR: a Community-Based Framework to Reduce Adult Substance Misuse, Intimate Partner Violence, Child Abuse, Suicidality, and Cumulative Risk. Prev Sci. 2020;21(7):949-59. Available from: <https://doi.org/10.1007/s11121-020-01156-w>. | Other patient population |
| Slesnick N, Zhang J, Feng X, Wu Q, Walsh L, Granello DH. Cognitive Therapy for Suicide Prevention: A Randomized Pilot with Suicidal Youth Experiencing Homelessness. Cognit Ther Res. 2019;44(2):402-11. Available from: <https://doi.org/10.1007/s10608-019-10068-1>. | Other patient population |
| Spirito A, Stanton C, Donaldson D, Boergers J. Treatment-as-usual for adolescent suicide attempters: implications for the choice of comparison groups in psychotherapy research. J Clin Child Adolesc Psychol. 2002;31(1):41-7. Available from: <https://doi.org/10.1207/S15374424JCCP3101_06>. | Wrong outcomes |
| Stanley IH, Hom MA, Sachs-Ericsson NJ, Gallyer AJ, Joiner TE. A pilot randomized clinical trial of a lethal means safety intervention for young adults with firearm familiarity at risk for suicide. J Consult Clin Psychol. 2020;88(4):372-83. Available from: <https://doi.org/10.1037/ccp0000481>. | Other patient population |
| Stecker T, Allan NP, Hoge C, Ashrafioun L, Conner KR. Efficacy of CBT for Treatment Seeking (CBT-TS) in Untreated Veterans and Service Members at Risk for Suicidal Behavior. J Gen Intern Med. 2023;38(12):2639-46. Available from: <https://doi.org/10.1007/s11606-023-08129-z>. | Other patient population |
| Su TP, Li CT, Lin WC, Wu HJ, Tsai SJ, Bai YM, et al. A Randomized, Double-Blind, Midazolam-Controlled Trial of Low-Dose Ketamine Infusion in Patients With Treatment-Resistant Depression and Prominent Suicidal Ideation. Int J Neuropsychopharmacol. 2023;26(5):331-9. Available from: <https://doi.org/10.1093/ijnp/pyad014>. | Other patient population |
| Szmulewicz A, Madenci A, Ferguson R, Liang MH, Lew R, Katz IR, Hernan MA. Estimating the per-protocol effect of lithium on suicidality in a randomized trial of individuals with depression or bipolar disorder. J Psychopharmacol. 2023;37(6):539-44. Available from: <https://doi.org/10.1177/02698811231166460>. | Other patient population |
| Tebbett-Mock AA, McGee M, Saito E. Efficacy and sustainability of dialectical behaviour therapy for inpatient adolescents: a follow-up study. Gen Psychiatr. 2021;34(4):e100452. Available from: <https://doi.org/10.1136/gpsych-2020-100452>. | Other study design |
| Torok M, Han J, McGillivray L, Wong Q, Werner-Seidler A, O'Dea B, et al. The effect of a therapeutic smartphone application on suicidal ideation in young adults: Findings from a randomized controlled trial in Australia. PLoS Med. 2022;19(5):e1003978. Available from: <https://doi.org/10.1371/journal.pmed.1003978>. | Other patient population |
| Udayan B. Effectiveness Of Repeated Ketamine Infusions On Suicidal Thoughts vs Ect: an Open Label Comparative Study. Indian journal of psychiatry. 2023;65:S56‐S7. | Conference abstract |
| Van Orden KA, Arean PA, Conwell Y. A Pilot Randomized Trial of Engage Psychotherapy to Increase Social Connection and Reduce Suicide Risk in Later Life. Am J Geriatr Psychiatry. 2021;29(8):789-800. Available from: <https://doi.org/10.1016/j.jagp.2021.03.009>. | Other patient population |
| Vieira F, Correia-Melo FS, Santos-Lima C, Souza-Marques B, Leal GC, Jesus-Nunes AP, et al. Ketamine and Esketamine augmentation for suicidal ideation: A randomized, double-blinded clinical trial. Gen Hosp Psychiatry. 2021;68:97-9. Available from: <https://doi.org/10.1016/j.genhosppsych.2020.12.011>. | Other patient population |
| Walton CJ, Bendit N, Baker AL, Carter GL, Lewin TJ. A randomised trial of dialectical behaviour therapy and the conversational model for the treatment of borderline personality disorder with recent suicidal and/or non-suicidal self-injury: An effectiveness study in an Australian public mental health service. Aust N Z J Psychiatry. 2020;54(10):1020-34. Available from: <https://doi.org/10.1177/0004867420931164>. | Other patient population |
| Waraan L, Rognli EW, Czajkowski NO, Mehlum L, Aalberg M. Efficacy of attachment-based family therapy compared to treatment as usual for suicidal ideation in adolescents with MDD. Clin Child Psychol Psychiatry. 2021;26(2):464-74. Available from: <https://doi.org/10.1177/1359104520980776>. | Other patient population |
| Wharff EA, Ginnis KB, Ross AM, White EM, White MT, Forbes PW. Family-Based Crisis Intervention With Suicidal Adolescents: A Randomized Clinical Trial. Pediatr Emerg Care. 2019;35(3):170-5. Available from: <https://doi.org/10.1097/PEC.0000000000001076>. | Other patient population |
| Wilkening J, Witteler F, Goya-Maldonado R. Suicidality and relief of depressive symptoms with intermittent theta burst stimulation in a sham-controlled randomized clinical trial. Acta Psychiatr Scand. 2022;146(6):540-56. Available from: <https://doi.org/10.1111/acps.13502>. | Other patient population |
| Wright-Hughes A, Graham E, Cottrell D, Farrin A. Routine hospital data - is it good enough for trials? An example using England's Hospital Episode Statistics in the SHIFT trial of Family Therapy vs. Treatment as Usual in adolescents following self-harm. Clin Trials. 2018;15(2):197-206. Available from: <https://doi.org/10.1177/1740774517751381>. | Data format not appropriate |
| Wu Q, Zhang J, Walsh L, Slesnick N. Family network satisfaction moderates treatment effects among homeless youth experiencing suicidal ideation. Behav Res Ther. 2020;125:103548. Available from: <https://doi.org/10.1016/j.brat.2019.103548>. | Other patient population |
| Wu Q, Zhang J, Walsh L, Slesnick N. Illicit Drug Use, Cognitive Distortions, and Suicidal Ideation Among Homeless Youth: Results From a Randomized Controlled Trial. Behav Ther. 2022;53(1):92-104. Available from: <https://doi.org/10.1016/j.beth.2021.06.004>. | Other patient population |
| Wu R, Zhong SY, Wang GH, Wu MY, Xu JF, Zhu H, et al. The Effect of Brief Mindfulness Meditation on Suicidal Ideation, Stress and Sleep Quality. Arch Suicide Res. 2023;27(2):215-30. Available from: <https://doi.org/10.1080/13811118.2021.1982800>. | Other patient population |
| Xavier A, Otero P, Blanco V, Vazquez FL. Efficacy of a problem-solving intervention for the indicated prevention of suicidal risk in young Brazilians: Randomized controlled trial. Suicide Life Threat Behav. 2019;49(6):1746-61. Available from: <https://doi.org/10.1111/sltb.12568>. | Data format not appropriate |
| Yang X, Liu D, Wang Y, Chen Y, Chen W, Yang C, et al. Effectiveness of Zhong-Yong thinking based dialectical behavior therapy group skills training versus supportive group therapy for lowering suicidal risks in Chinese young adults: A randomized controlled trial with a 6-month follow-up. Brain Behav. 2020;10(6):e01621. Available from: <https://doi.org/10.1002/brb3.1621>. | Other patient population |
| Yen S, Ranney ML, Krek M, Peters JR, Mereish EH, Tezanos KM, et al. Skills to Enhance Positivity in Suicidal Adolescents: Results from a Pilot Randomized Clinical Trial. J Posit Psychol. 2020;15(3):348-61. Available from: <https://doi.org/10.1080/17439760.2019.1615105>. | Data format not appropriate |
| Yen S, Spirito A, Weinstock LM, Tezanos K, Kolobaric A, Miller I. Coping long term with active suicide in adolescents: Results from a pilot randomized controlled trial. Clin Child Psychol Psychiatry. 2019;24(4):847-59. Available from: <https://doi.org/10.1177/1359104519843956>. | Data format not appropriate |
| Zhang D, Tian Y, Wang R, Wang L, Wang P, Su Y. Effectiveness of a resilience-targeted intervention based on "I have, I am, I can" strategy on nursing home older adults' suicidal ideation: A randomized controlled trial. J Affect Disord. 2022;308:172-80. Available from: <https://doi.org/10.1016/j.jad.2022.04.046>. | Other patient population |
| Zhou Y, Wang C, Lan X, Zheng W, Li H, Chao Z, et al. The effectiveness of repeated intravenous ketamine on subjective and objective psychosocial function in patients with treatment-resistant depression and suicidal ideation. J Affect Disord. 2022;304:78-84. Available from: <https://doi.org/10.1016/j.jad.2022.02.032>. | Other patient population |
| Zullo L, King J, Nakonezny PA, Kennard BD, Emslie G, Stewart SM. Implementing the interpersonal theory of suicide to improve outcomes in suicidal adolescents: A pilot trial. Suicide Life Threat Behav. 2021;51(4):633-40. Available from: <https://doi.org/10.1111/sltb.12745>. | Other study design |
| Zullo L, van Dyk IS, Ollen E, Ramos N, Asarnow J, Miranda J. Treatment recommendations and barriers to care for suicidal LGBTQ youth: A quality improvement study. Evid Based Pract Child Adolesc Ment Health. 2021;6(3):393-409. Available from: <https://doi.org/10.1080/23794925.2021.1950079>. | Other study design |
