# Supplementary figures and images for: Effects of interventions for self-harm in children and adolescents: a systematic review and meta-analysis
Source: Eur Child Adolesc Psychiatry. 2025 Sep 27;35(1):91–107. doi: 10.1007/s00787-025-02859-7 (PMC12916994; doi:10.1007/s00787-025-02859-7)

Table S2. Risk of bias in the included studies


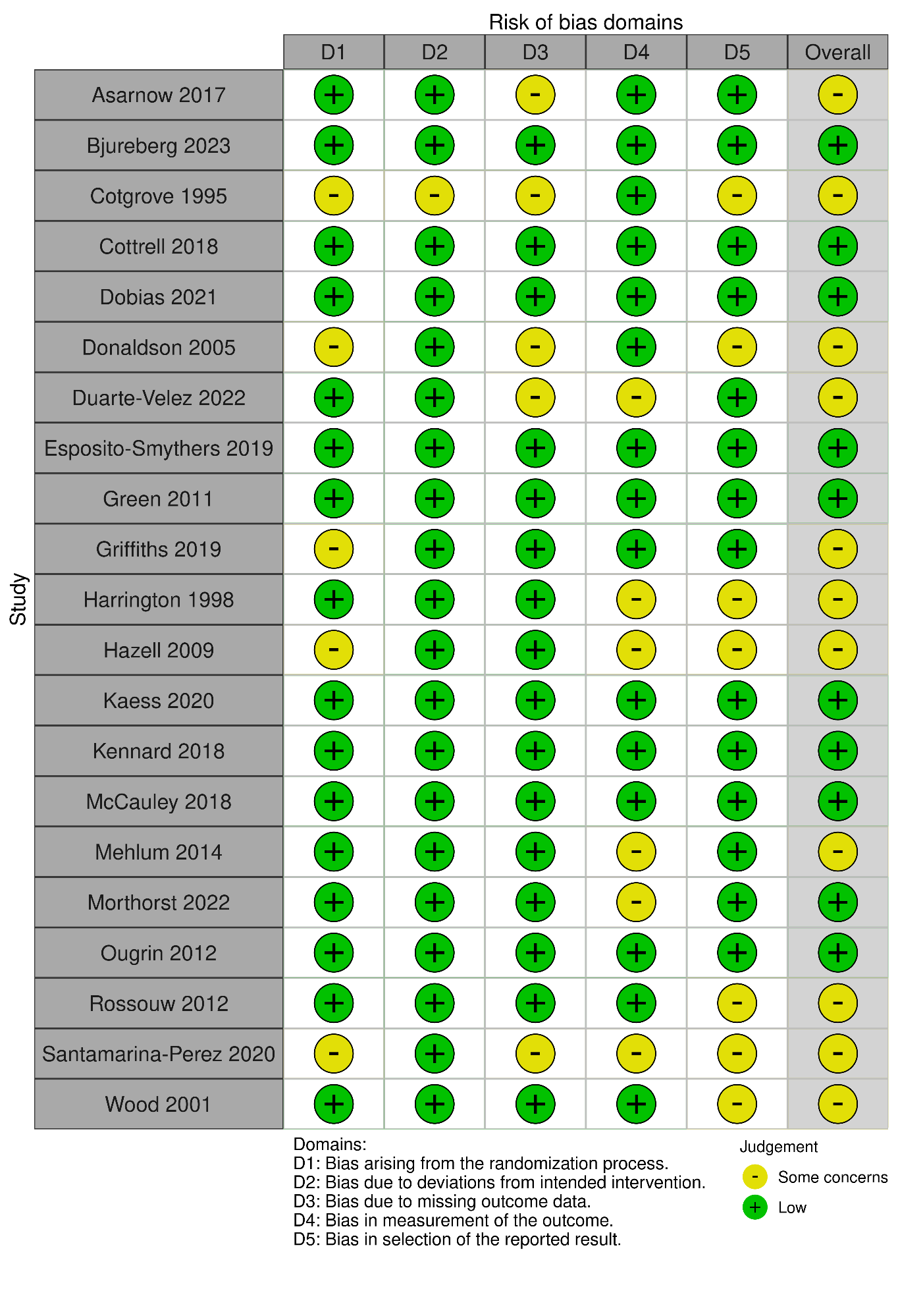

Supplement: Supplementary file 3 — (DOCX 347 KB) [file 787_2025_2859_MOESM3_ESM.docx]
