# Supplementary material for: Effects of interventions for self-harm in children and adolescents: a systematic review and meta-analysis
Source: Eur Child Adolesc Psychiatry. 2025 Sep 27;35(1):91–107. doi: 10.1007/s00787-025-02859-7 (PMC12916994; doi:10.1007/s00787-025-02859-7)
Supplement: Supplementary file 4 — (DOCX 553 KB) [file 787_2025_2859_MOESM4_ESM.docx]

# Supplement 2 Metaanalyses and Summary of Findings tables

### Contents

[Appendix 4 Metaanalyses and Summary of Findings tables 1](#_Toc182841452)

[Contents 1](#_Toc182841453)

[Cognitive behavioral therapy (CBT) 2](#_Toc182841454)

[Dialectical Behavior Therapy for Adolescents (DBT-A) 8](#_Toc182841455)

[Internet-delivered emotion regulation individual therapy (IERITA) 13](#_Toc182841456)

[Mentalization-Based Treatment for Adolescents (MBT-A) 16](#_Toc182841457)

[Motivational interviewing 19](#_Toc182841458)

[Brief Admission by Self-Referral 21](#_Toc182841459)

[Group therapy versus TAU 22](#_Toc182841460)

[Family therapy 26](#_Toc182841461)

[References 28](#_Toc182841462)

### Cognitive behavioral therapy (CBT)

#### Metaanalyses

**Figure S1** SA in last six months at post-intervention (3–6 months post-allocation)*.


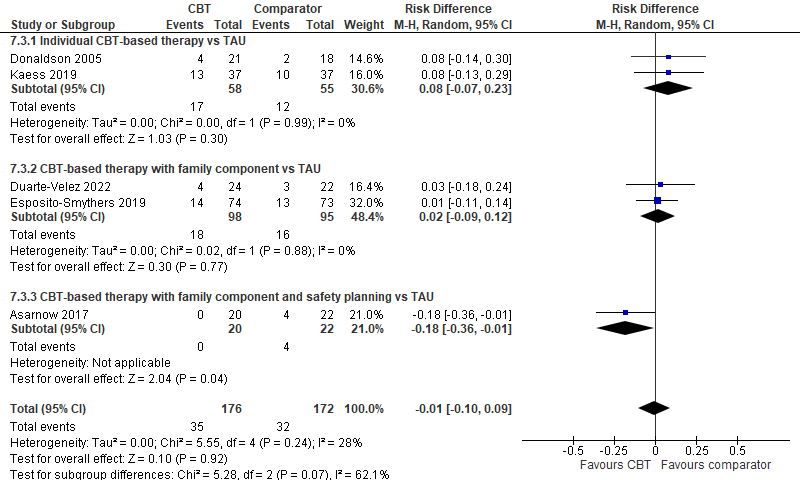


*Data from Kaess 2019 has been received upon request

**SA** = Suicide Attempts

**Figure S2** SA in last six months at 10–12 months post-allocation*.


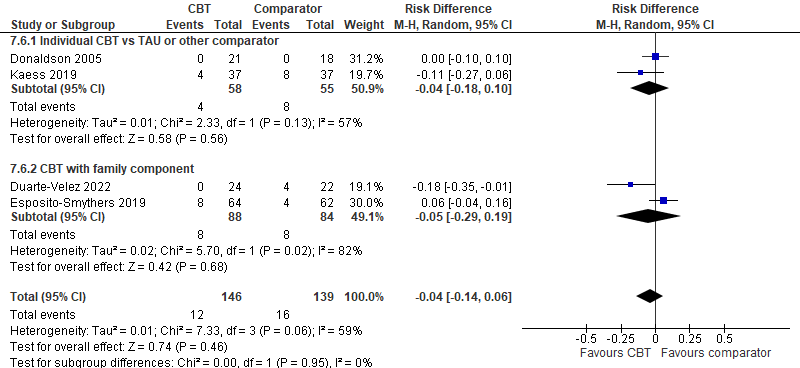


*Data from Kaess 2019 has been received upon request

**SA** = Suicide Attempts

**Figure S3** NSSI during last month, at post-intervention (4–6 months post-allocation)*.


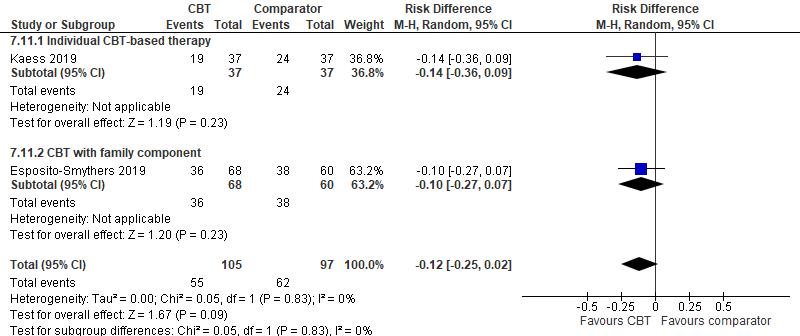


*Data from Kaess 2019 has been received upon request

**NSSI** = Non-Suicidal Self-Injury

**Figure S4** Suicide ideation scores* at post-intervention.


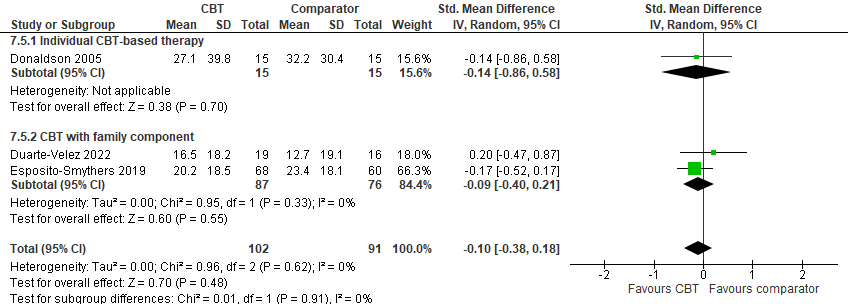


*(Donaldson: SIQ, Duarte-Velez and Esposito-Smythers: SIQ-JR)

**SIQ** = Suicidal Ideation Questionnaire (range 0–180); **SIQ-JR** = Suicidal Ideation Questionnaire, Junior Version (range 0–90)

**Figure S5** Suicide ideation scores at 10–12 months post-allocation.


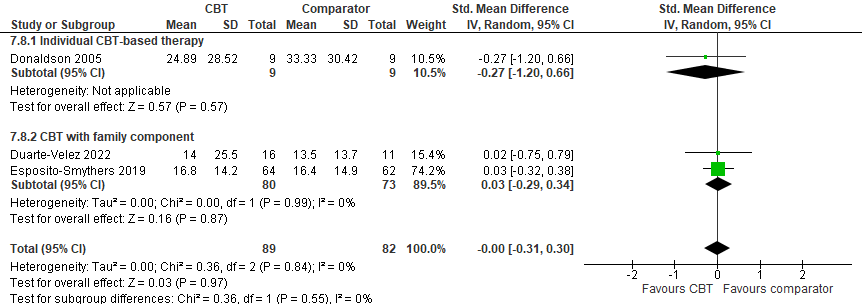


**Figure S6** Depression scores* post-intervention.


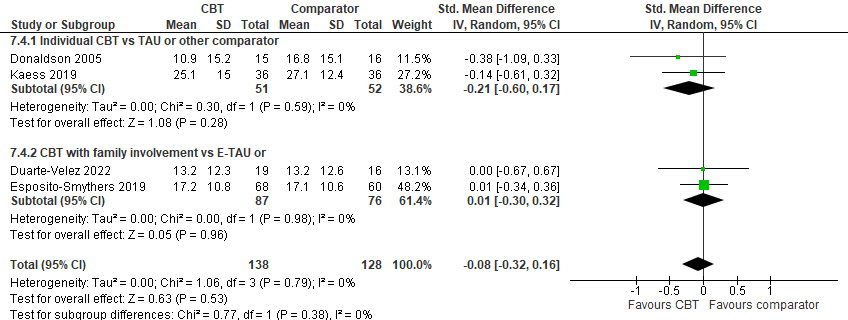


*Kaess 2019: BDI-II, Donaldsson: CES-D, Duarte-Velez 2022: CDI-II, Esposito-Smythers 2019: CDI-II

**BDI-II** = Beck-Depression-Inventory-II (range 0-63); **CDI-II** = Children’s Depression Inventory-2nd Edition (range 0–56); **CES-D** = The Center for Epidemiologic Studies-Depression Scale (range 0-60)

**Figure S7** Depression scores* at 10–12 months post-allocation.


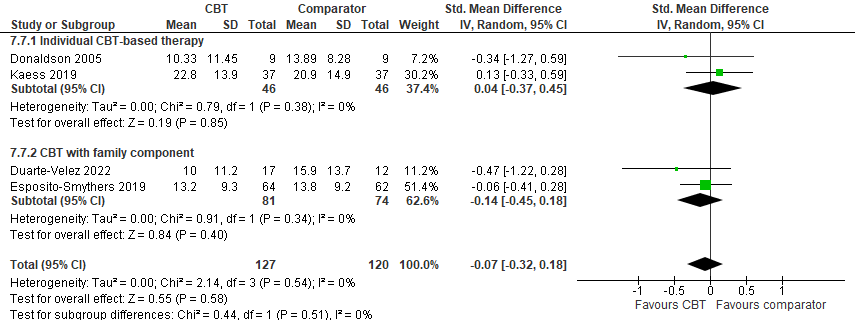


*Kaess 2019: BDI-II, Donaldsson: CES-D, Duarte-Velez 2022: CDI-II, Esposito-Smythers 2019: CDI-II

**BDI-II** = Beck-Depression-Inventory-II (range 0-63); **CDI-II** = Children’s Depression Inventory-2nd Edition (range 0–56); **CES-D** = The Center for Epidemiologic Studies-Depression Scale (range 0-60)

#### Table S3. Summary of findings for CBT-based therapy versus TAU

| Outcome | Number of participants  (Number of studies)  References | Absolute effect  (95% CI) | Certainty of the evidence (GRADE) | Downrating (GRADE) | Comment |
| --- | --- | --- | --- | --- | --- |
| **Number of participants with suicide attempts at post-intervention** | 348  (5 RCT)  [1-5] | RD= –0.01 (–0.10 to 0.09) | ⊕⊕⊖⊖  Low | Imprecision^1^ | Evidence suggests CBT results in little to no difference |
| **Number of participants with suicide attempts at 10–12 months post-allocation** | 285  (4 RCT)  [2-5] | RD= –0.04 (–0.14 to 0.06) | ⊕⊕⊖⊖  Low | Imprecision^1^ | Evidence suggests CBT results in little to no difference |
| **Number of participants with suicidal attempts at 18 months post-allocation** | 124  (1 RCT)  [4] | RD=0.06 (–0.02 to 0.15) | ⊕⊖⊖⊖  Very low | Imprecision^2^ |  |
| **Number of participants with suicidal attempts at 2-4 years post-allocation** | 74  (1 RCT  [6] | RD=0.05 (–0.14 to 0.03) | ⊕⊖⊖⊖  Very low | Imprecision^2^ | Follow-up study of Kaess 2019, data were received upon request |
| **Number of participants with NSSI at post-intervention** | 202  (2 RCT)  [4, 5] | RD= –0.12 (–0.25 to 0.02) | ⊕⊖⊖⊖  Very low | Imprecision^2^ |  |
| **Number of participants with NSSI at follow-up** | 124  (1 RCT)  [4] | At 12 months:  RD= –0.09 (–0.25 to 0.07)  At 18 months: RD=0.06 (–0.08 to 0.20) | ⊕⊖⊖⊖  Very low | Imprecision^2^ |  |
| **Number of participants with NSSI at 2-4 years post-allocation** | 74  (1 RCT)  [6] | RD=0.03 (–0.16 to 0.21) | ⊕⊖⊖⊖  Very low | Imprecision^2^ | Follow-up study of Kaess 2019, data were received upon request |
| **Completed suicides** | 31  (1 RCT)  [2] | KBT: 0  TAU: 0 | ⊕⊖⊖⊖  Very low | Imprecision^3^ | Metaanalysis not performed because of 0 events |
| **Suicidal ideation (SIQ or SIQ-JR), at post-intervention** | 193  (3 RCT)  [2-4] | SMD= –0.10 (–0.38 to 0.18) | ⊕⊖⊖⊖  Very low | Imprecision^2^ |  |
| **Suicidal ideation (SIQ or SIQ-JR), at 10–12 months post-allocation** | 171  (3 RCT)  [2-4] | SMD= –0.00 (–0.31 to 0.30) | ⊕⊖⊖⊖  Very low | Imprecision^2^ |  |
| **Suicidal ideation (SIQ or SIQ-JR), at 18 months post-allocation** | 124  (1 RCT)  [4] | MD=2.40 (–1.30 to 6.10) | ⊕⊖⊖⊖  Very low | Imprecision^2^ |  |
| **Depression (BDI-II, CES-D, CDI-II), at post-intervention** | 266  (4 RCT)  [2-5] | SMD= –0.08 (–0.32 to 0.16) | ⊕⊖⊖⊖  Very low | Imprecision^2^ |  |
| **Depression (BDI-II, CES-D, CDI-II), at 10-12 months post-allocation** | 247  (4 RCT)  [2-5] | SMD= –0.07 (–0.32 to 0.18) | ⊕⊖⊖⊖  Very low | Imprecision^2^ |  |
| **Depression (CDI-II), at 18 months post-allocation** | 124  (1 RCT)  [4] | MD=5.30 (1.95 to 8.65) | ⊕⊖⊖⊖  Very low | Imprecision^2^ |  |
| **Anxiety** | 0  (0 RCT) | - | - | - | Outcome not reported in any study |
| **General function** | 0  (0 RCT) | - | - | - | Outcome not reported in any study |

**BDI-II** = Beck-Depression-Inventory-II (range 0-63); **BSSI** = Beck Scale for Suicide Ideation (range 0–38); **CDI-II** = Children’s Depression Inventory-2nd Edition (range 0–56); **CES-D** = The Center for Epidemiologic Studies-Depression Scale (range 0-60); **CI** = Confidence Interval; **GRADE** = The Grading of Recommendations Assessment, Development and Evaluation; **MD** = Mean Difference; **NSSI** = Non-Suicidal Self-Injury; **RCT** = Randomized controlled trial; **RD** = Risk Difference; **SIQ** = Suicidal Ideation Questionnaire (range 0–180); **SIQ-JR** = Suicidal Ideation Questionnaire, Junior Version (range 0–90); **SMD** = Standardized Mean Difference; **TAU** = Treatment As Usual

^1^ Downrated –2 because of imprecision: few participants

^2^ Downrated –3 because of imprecision: non-significant results and few participants

^3^ Downrated –3 because of imprecision: few participants and very few events

### Dialectical Behavior Therapy for Adolescents (DBT-A)

#### Metaanalyses

**Figure S8** Number of participants with self-harm* at post-intervention.


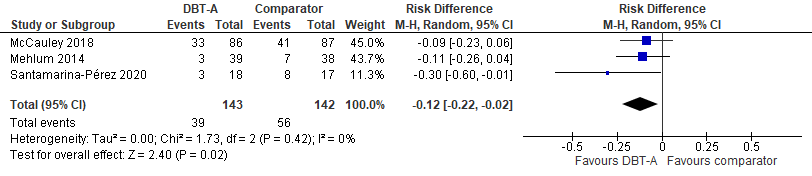


*Mehlum 2014: self-harm leading to hospital/ED visit during the trial (last 19 weeks); McCauley 2018: self-harm (any, NSSI or SA) during trial/ last 6 months; Santa-Maria Perez: NSSI in last 4 weeks

**NSSI** = Non-Suicidal Self-Injury

**Figure S9** Frequency of self-harm* at post-intervention.


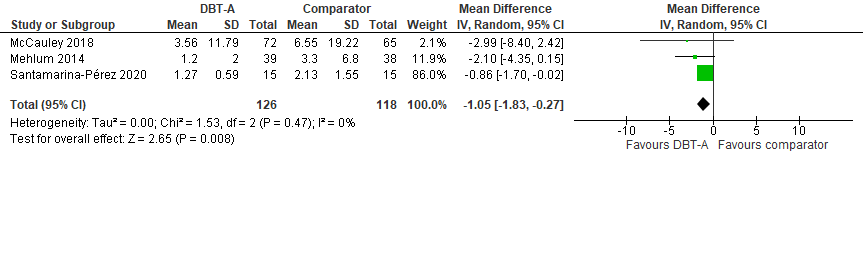
*Santa-Maria Perez 2020: NSSI last 4 weeks; McCauley 2018: Number of self-harm episodes in last 6 months (from baseline to end of study)

**NSSI** = Non-Suicidal Self-Injury

**Figure S10** Number of participants with suicide attempts at post-intervention.


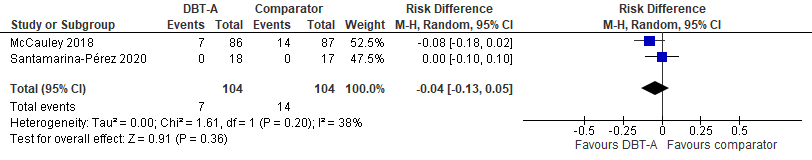


**Figure S11** Number of participants with suicide attempts at 12–19 months* post-allocation.


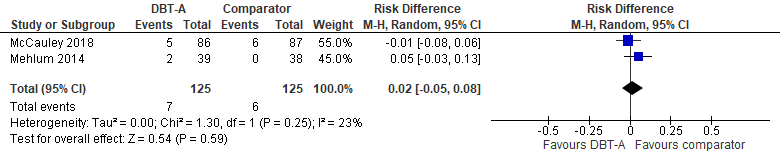


Mehlum 2014: 19 months (71 weeks) post-allocation; McCauly 2018: 12 months post-allocation

**Figure S12** Suicidal ideation scores (SIQ-JR) at post-intervention.


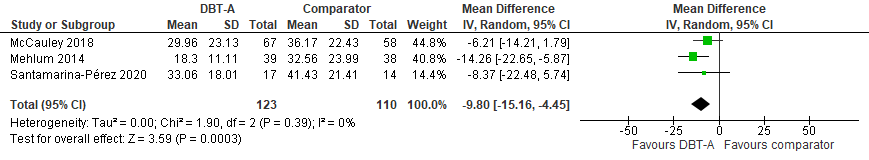


**SIQ-JR** = Suicidal Ideation Questionnaire, Junior Version (range 0–90)

**Figure S13** Suicidal ideation (SIQ-JR) at 12–19 months post-allocation.


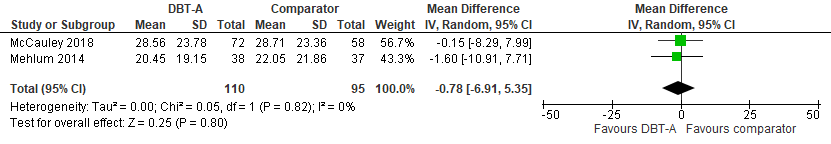


**SIQ-JR** = Suicidal Ideation Questionnaire, Junior Version (range 0–90)

**Figure S14** Depression symptoms* at post-intervention.


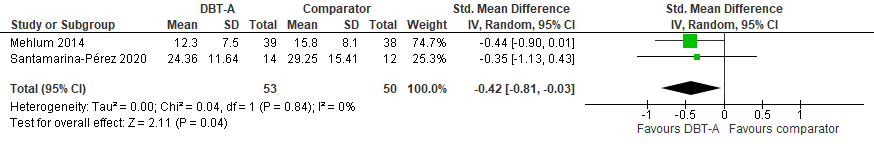


*Mehlum 2014: MADRS; Santamarina-Perez 2020: BDI-II

**BDI-II** = Beck-Depression-Inventory-II (range 0-63); **MADRS** = (Montgomery-Åsberg Depression Rating Scale (range 0–60)

**Figure S15** General function scores (C-GAS) at post-intervention.


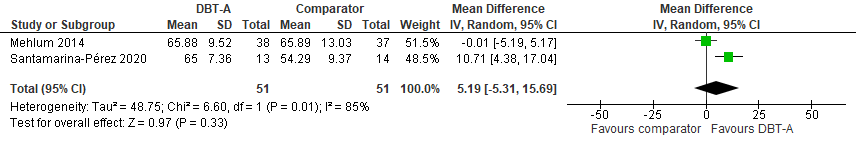


**C-GAS** = Children's Global Assessment Scale (range 1–100)

#### Table S4. Summary of findings for DBT-A versus alternative psychotherapy

| Outcome | Number of participants  (Number of studies)  References | Absolute effect  (95% CI) | Certainty of the evidence (GRADE) | Downrating (GRADE) | Comment |
| --- | --- | --- | --- | --- | --- |
| **Number of participants with self-harm at post-intervention** | 285  (3 RCT)  [7-9] | RD= –0.12 (-0.22 to –0.02) | ⊕⊕⊕⊖  Moderate | Imprecision^1^ | DBT-A probably reduces outcome |
| **Number of participants with self-harm at 12 months post-allocation** | 173  (1 RCT)  [7] | RD= –0.03 (–0.17 to –0.11) | ⊕⊖⊖⊖  Very low | Imprecision^2^ |  |
| **Frequency of self-harm at post-intervention** | 244  (3 RCT)  [7-9] | MD= –1.05 (–1.83 to –0.27) | ⊕⊕⊕⊖  Moderate | Imprecision^1^ | DBT-A probably reduces outcome |
| **Frequency of self-harm at 12-19 months post-allocation** | 202  (2 RCT)  [7, 10] | MD= –9.30 (–14.00 to –4.60) Mehlum 2016  OR=0.60 (0.24 to 1.52) McCauley 2018 | ⊕⊖⊖⊖  Very low | Imprecision^2^ | Metaanalysis not performed since data were reported on different formats |
| **Frequency of self-harm at 3 years post-allocation** | 71  (1 RCT)  [11] | MD=12.62 (–1.76 to 27.00) | ⊕⊖⊖⊖  Very low | Imprecision^2^ |  |
| **Number of participants with suicide attempts at post-intervention** | 208  (2 RCT)  [7, 9] | RD= –0.04 (–0.13 to 0.05) | ⊕⊖⊖⊖  Very low | Imprecision^2^ |  |
| **Number of participants with suicide attempts at 12-19 months post-allocation** | 250  (2 RCT)  [7, 11] | RD=0.02 (–0.05 to 0.08) | ⊕⊖⊖⊖  Very low | Imprecision^2^ |  |
| **Number of participants with suicide attempts at 3 years post-allocation** | 77  (1 RCT)  [11] | RD= –0.11 (–0.24 to 0.03) | ⊕⊖⊖⊖  Very low | Imprecision^2^ |  |
| **Completed suicides** | 285  (3 RCT) | DBT-A: 0  ComparatOR=1 | ⊕⊖⊖⊖  Very low | Imprecision^3^ | Metaanalysis not performed because of very few events |
| **Suicidal ideation (SIQ-JR), at post-intervention** | 233  (3 RCT)  [7-9] | MD= –9.80 (–15.16 to –4.45) | ⊕⊕⊕⊖  Moderate | Imprecision^1^ | DBT-A probably reduces outcome |
| **Suicidal ideation (SIQ-JR), at 12-19 months post-allocation** | 205  (2 RCT)  [7, 10] | MD= –0.78 (–6.91 to 5.35) | ⊕⊖⊖⊖  Very low | Imprecision^2^ |  |
| **Suicidal ideation (SIQ-JR), at 3 years post-allocation** | 71  (1 RCT)  [11] | MD= –3.51 (–12.04 to 5.02) | ⊕⊖⊖⊖  Very low | Imprecision^2^ |  |
| **Depression (MADRS or BDI-II), at post-intervention** | 103  (2 RCT)  [8, 9] | SMD= –0.42 (–0.81 to –0.03) | ⊕⊕⊖⊖  Low | Imprecision^4^ | DBT-A may reduce outcome |
| **Depression (MADRS), at 19 months post-allocation** | 75  (1 RCT)  [10] | MD= –0.64 (–4.53 to 3.25) | ⊕⊖⊖⊖  Very low | Imprecision^2^ |  |
| **Depression (MADRS), at 3 years post-allocation** | 71  (1 RCT)  [11] | MD=1.36 (–1.96 to 4.68) | ⊕⊖⊖⊖  Very low | Imprecision^2^ |  |
| **Anxiety** | 0  (0 RCT) | - | - | - | Outcome not reported in any study |
| **General function (C-GAS), at post-intervention** | 102  (2 RCT)  [8, 9] | MD=5.19 (–5.31 to 15.69) | ⊕⊖⊖⊖  Very low | Imprecision^2^ |  |
| **General function (C-GAS), at 19 months post-allocation** | 75  (1 RCT)  [10] | MD=1.50 (–4.40 to 7.40) | ⊕⊖⊖⊖  Very low | Imprecision^2^ |  |
| **General function (C-GAS), at 3 years post-allocation** | 71  (1 RCT)  [11] | MD= –1.15 (–6.49 to 4.19) | ⊕⊖⊖⊖  Very low | Imprecision^2^ |  |

**BDI-II** = Beck-Depression-Inventory-II (range 0-63); **C-GAS** = Children's Global Assessment Scale (range 1–100); **CI** = Confidence Interval; **GRADE** = The Grading of Recommendations Assessment, Development and Evaluation; **MADRS** = (Montgomery-Åsberg Depression Rating Scale (range 0–60); **MD** = Mean Difference; **RCT** = Randomized controlled trial; **RD** = Risk Difference; **SIQ-JR** = Suicidal Ideation Questionnaire, Junior Version (range 0–90); **SMD** = Standardized Mean Difference

^1^ Downrated –1 because of precision: few participants

^2^ Downrated –3 because of precision: non-significant results and few participants

^3^ Downrated –3 because of precision: few participants and very few events

^4^ Downrated –2 because of precision: few participants

### Internet-delivered emotion regulation individual therapy (IERITA)

#### Metaanalyses

**Figure S16** Number of participants with NSSI* at post-intervention**.


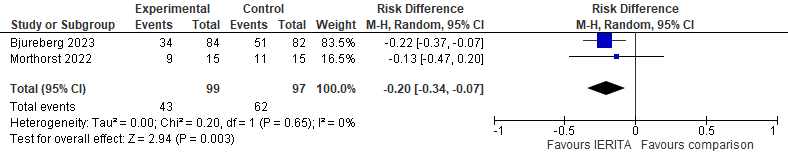


*Bjureberg 2023: Clinicians-rated version of DSHI-Y; Morthorst 2022: Self-reported DSHI-Y. Data from Bjureberg 2023 were received upon request.

** Bjureberg 2023: Outcome was assessed 1 month post-treatment; Morthorst 2022: Outcome was assessed post-treatment. Treatment length was 12 weeks in both studies.

**DSHI-Y** = Deliberate Self Harm Inventory - Youth version; **NSSI** = Non-Suicidal Self-Injury

**Figure S17** Frequency of NSSI episodes* at post-intervention.


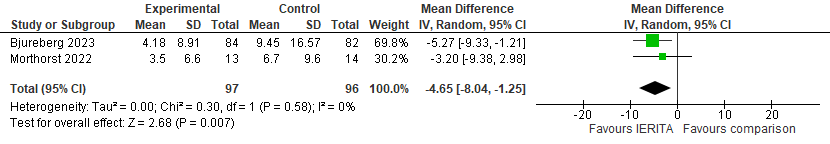


*Number of NSSI episodes within the last 4 weeks: Bjureberg 2023: clinicians-rated version of DSHI-Y; Morthorst 2022: self-reported DSHI-Y

**DSHI-Y** = Deliberate Self Harm Inventory - Youth version; **NSSI** = Non-Suicidal Self-Injury

**Figure S18** Depression scores (DASS-21 subscale depression) at post-intervention*.


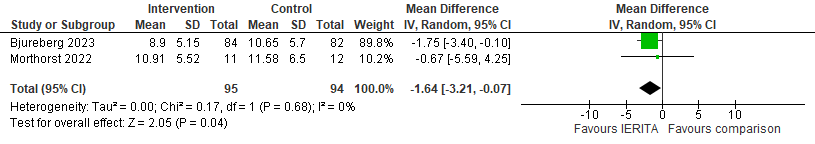


*Data from Bjureberg 2023 were received upon request.

**DASS-21** = Depression, Anxiety and Stress Scale, 21 items (range 0–42)

**Figure S19** Anxiety scores (DASS-21 subscale anxiety) at post-intervention*.


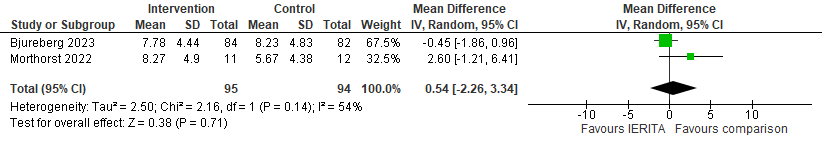


*Data from Bjureberg 2023 were received upon request.

**DASS-21** = Depression, Anxiety and Stress Scale, 21 items (range 0–42)

#### Table S5. Summary of findings for IERITA versus TAU.

| Outcome | Number of participants  (Number of studies)  References | Absolute effect  (95% CI) | Certainty of the evidence (GRADE) | Downrating (GRADE) | Comment |
| --- | --- | --- | --- | --- | --- |
| **Number of participants with NSSI at post-intervention** | 196  (2 RCT)  [12, 13] | RD= –0.20 (–0.34 to –0.07) | ⊕⊕⊖⊖  Low | Imprecision^1^ | IERITA may reduce outcome |
| **Frequency of NSSI at post-intervention** | 193  (2 RCT)  [12, 13] | MD= –4.65 (–8.04 to -1.25) | ⊕⊕⊖⊖  Low | Imprecision^1^ | IERITA may reduce outcome |
| **Frequency of NSSI at 6 months post-allocation** | 166  (1 RCT)  [12] | MD= –1.47 (–3.51 to 0.57) | ⊕⊖⊖⊖  Very low | Imprecision^2^ |  |
| **Number of participants with suicide attempts at post-intervention** | 166  (1 RCT)  [12] | RD= –0.05 (–0.13 to 0.03) | ⊕⊖⊖⊖  Very low | Imprecision^2^ |  |
| **Completed suicides** | 0  (0 RCT) | - | - | - | Outcome not reported in any study |
| **Suicidal ideation** | 0  (0 RCT) | - | - | - | Outcome not reported in any study |
| **Depression (DASS-21 subscale), at post-intervention** | 189  (2 RCT)  [12, 13] | MD= –1.64 (–3.21 to -0.07) | ⊕⊕⊖⊖  Low | Imprecision^2^ | IERITA may reduce outcome |
| **Depression (DASS-21 subscale), at 6 months post-allocation** | 166  (1 RCT)  [12] | MD= –0.69 (–2.50 to 1.12) | ⊕⊖⊖⊖  Very low | Imprecision^2^ |  |
| **Anxiety (DASS-21 subscale), at post-intervention** | 189  (2 RCT)  [12, 13] | MD=0.54 (–2.26 to 3.34) | ⊕⊖⊖⊖  Very low | Imprecision^2^ |  |
| **Anxiety (DASS-21 subscale), at 6 months post-allocation** | 166  (1 RCT)  [12] | MD=–0.18 (–1.63 to 1.27) | ⊕⊖⊖⊖  Very low | Imprecision^2^ |  |
| **General function (C-GAS), at post-intervention** | 159  (1 RCT)  [12] | MD=1.98 (–0.71 to 4.67) | ⊕⊖⊖⊖  Very low | Imprecision^2^ |  |

**C-GAS** = Children's Global Assessment Scale (range 1–100); **CI** = Confidence Interval; **DASS-21** = Depression, Anxiety and Stress Scale, 21 items (range 0–42); **GRADE** = The Grading of Recommendations Assessment, Development and Evaluation; **MD** = Mean Difference; **NSSI** = Non-Suicidal Self-Injury; **RCT** = Randomized controlled trial; **RD** = Risk Difference; **TAU** = Treatment As Usual

^1^ Downrated –2 because of imprecision: few participants

^2^ Downrated –3 because of imprecision: non-significant results and few participants

### Mentalization-Based Treatment for Adolescents (MBT-A)

#### Metaanalyses

**Figure S20** Number of participants with self-harm* at post-intervention**.


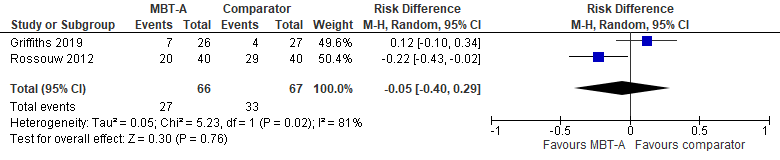


*Length of intervention: Rossouw 2012: 12 months; Griffith 2019:12 weeks.

**Griffith: data on self-harm with ED presentation self-harm were received from author upon request. Rossouw: self-reported self-harm from RT from RTSHI questionnaire.

**ED** = Emergency Department; **RTSHI** = Risk-Taking and Self-Harm Inventory

**Figure S21** Number of participants with self-harm at 3 months treatment.


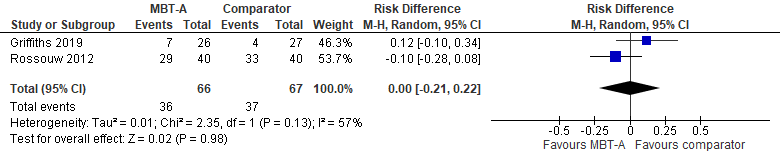


**Figure S22** Number of participants with self-harm at 9 months post-allocation.


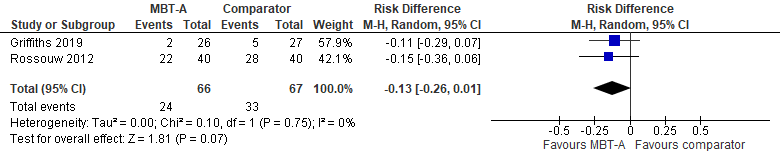


**Figure S23** Depression scores at post-intervention.

**
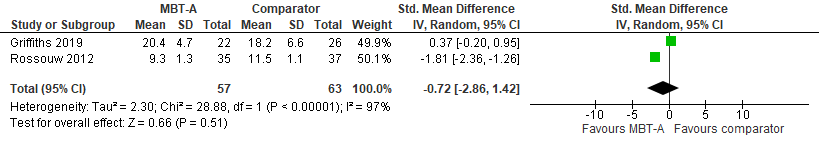
**

*Rossouw: MFQ; Griffiths: RCADS MD

**MD** = Mean Difference; **MFQ** = Mood and Feelings Questionnaire (range 0–26); **RCADS MD** = Revised Child Anxiety and Depression Scale, Major Depression subscale (range 0–30)

**Figure S24** Anxiety scores post-intervention*.

**
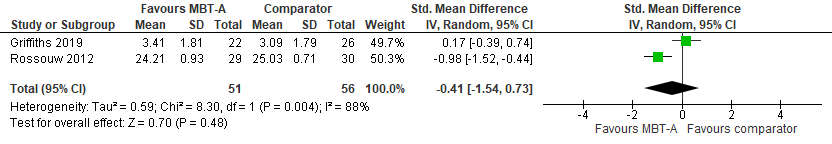
**

*Rossouw: ECRS anxiety subscale; Griffiths, ECRS revised Child version, anxiety subscale.

**ECRS** = Experiences in Close Relationships Scale

#### Table S6. Summary of findings for MBT-A versus TAU.

| Outcome | Number of participants  (Number of studies)  ref | Absolute effect  (95% CI) | Certainty of the evidence (GRADE) | Downrating (GRADE) | Comment |
| --- | --- | --- | --- | --- | --- |
| **Number of participants with self-harm** | 133  (2 RCT)  [14, 15] | At post-intervention:  RD= –0.05 (–0.40 to 0.29)  At 3 months post-allocation:  RD=0.00 (–0.20 to 0.21)  At 8–9 months post-allocation:  RD= –0.13 (–0.26 to 0.01) | ⊕⊖⊖⊖  Very low | Imprecision, Inconsistency^1^ |  |
| **Suicide attempts** | 0  (0 RCT) | - | - | - | Outcome not reported in any study |
| **Completed suicides** | 133  (2 RCT) | MBT-A: 0  TAU: 0 | ⊕⊖⊖⊖  Very low | Imprecision^2^ | Metaanalysis not performed because of 0 events |
| **Suicidal ideation** | 0  (0 RCT) | - | - | - | Outcome not reported in any study |
| **Depression scores (MFQ and RADSC MD) at post-intervention** | 120  (2 RCT)  [14, 15] | SMD= –0.72 (–2.86 to 1.42) | ⊕⊖⊖⊖  Very low | Imprecision^3^ |  |
| **Anxiety (ECRS and ECRS Child version, subscales for anxiety) at post-intervention** | 107  (2 RCT)  [14, 15] | SMD= –0.41 (–1.54 to 0.73) | ⊕⊖⊖⊖  Very low | Imprecision^3^ |  |
| **General function** | 0  (0 RCT) | - | - | - | Outcome not reported in any study |

**CI** = Confidence Interval; **ECRS** = Experiences in Close Relationships Scale; **GRADE** = The Grading of Recommendations Assessment, Development and Evaluation; **MFQ** = Mood and Feelings Questionnaire (range 0–26); **RCT** = Randomized controlled trial; **RD** = Risk Difference; **SMD** = Standardized Mean Difference; **TAU** = Treatment As Usual

^1^ Downrated –2 because of imprecision: non-significant results (CI includes both important benefit and harm), and -1 because of inconsistency: the results of the three studies are substantially different

^2^ Downrated –3 because of imprecision: very few events and few participants

^3^ Downrated –3 because of imprecision: non-significant results and few participants

**Brief interventions**

#### Table S7. Summary of findings for As Safe as Possible (ASAP) versus TAU.

| Outcome | Number of participants  (Number of studies)  Reference | Absolute effect  (95% CI) | Certainty of the evidence (GRADE) | Downrating (GRADE) | Comment |
| --- | --- | --- | --- | --- | --- |
| **Number of participants with NSSI, at 6 months post-allocation** | 66  (1 RCT)  [16] | RD=0.01 (–0.23 to 0.24) | ⊕⊖⊖⊖  Very low | Imprecision^1^ |  |
| **Number of participants with suicide attempts, at 6 months post-allocation** | 66  (1 RCT)  [16] | RD= –0.13 (–0.33 to 0.06) | ⊕⊖⊖⊖  Very low | Imprecision^1^ |  |
| **Completed suicides** | 0  (0 RCT) | - | - | - | Outcome not reported in study |
| **Number of participants with uicidal ideation (SIQ-JR)** | 66  (1 RCT)  [16] | RD= –0.07 (–0.30 to 0.16) | ⊕⊖⊖⊖  Very low | Imprecision^1^ |  |
| **Depression** | 0  (0 RCT) | - | - | - | Outcome not reported in study |
| **Anxiety** | 0  (0 RCT) | - | - | - | Outcome not reported in study |
| **General function** | 0  (0 RCT) | - | - | - | Outcome not reported in study |

**CI** = Confidence Interval; **GRADE** = The Grading of Recommendations Assessment, Development and Evaluation; **NSSI** = Non-Suicidal Self-Injury; **RCT** = Randomized controlled trial; **RD** = Risk Difference; **SIQ-JR** = Suicidal Ideation Questionnaire, Junior Version (range 0–90); **TAU** =Treatment As Usual

^1^ Downrated –3 because of imprecision: non-significant results, few participants and only one study

#### Table S8. Summary of findings for Therapeutic Assessment (TA) versus Assessment As Usual (AAU).

| Outcome | Number of participants  (Number of studies)  Reference | Absolute effect  (95% CI) | Certainty of the evidence (GRADE) | Downrating (GRADE) | Comment |
| --- | --- | --- | --- | --- | --- |
| **Number of participants with self-harm leading to hospital admission, at 2 years post-allocation** | 70  (1 RCT)  [17] | RD= –0.06 (–0.25 to 0.14) | ⊕⊖⊖⊖  Very low | Imprecision^1^ | Outcome not reported at earlier timepoints |
| **Suicide attempts** | 0  (0 RCT) | - | - | - | Outcome not reported in study |
| **Completed suicides** | 0  (0 RCT) | TA: 0  AAU: 0 | ⊕⊖⊖⊖  Very low | Imprecision^1^ |  |
| **Suicidal ideation** | 0  (0 RCT) | - | - | - | Outcome not reported in study |
| **Depression** | 0  (0 RCT) |  | - |  | Outcome not reported in study |
| **Anxiety** | 0  (0 RCT) | - | - | - | Outcome not reported in study |
| **General function, at 3 months post-allocation**  **(C-GAS)** | 70  (1 RCT)  [17] | MD=4.49 (−0.98 to 9.96) | ⊕⊖⊖⊖  Very low | Imprecision^1^ |  |

**AAU** = Assessment As Usual; **C-GAS** = Children's Global Assessment Scale (range 1–100); **CI** = Confidence Interval; **GRADE** = The Grading of Recommendations Assessment, Development and Evaluation; **MD** = Mean Difference; **RCT** = Randomized controlled trial; **RD** = Risk Difference

^1^ Downrated –3 because of imprecision: non-significant results, few participants an only one study

^2^ Downrated –3 because of imprecision: very few events and few participants

### Brief Admission by Self-Referral

#### Table S9. Summary of findings for Brief Admission by Self-Referral versus TAU.

| Outcome | Number of participants  (Number of studies)  Reference | Absolute effect  (95% CI) | Certainty of the evidence (GRADE) | Downrating (GRADE) | Comment |
| --- | --- | --- | --- | --- | --- |
| **Self-harm** | 0  (0 RCT) | - | - | - | Outcome not reported in study |
| **Number of participants with suicide attempts, at 1 year post-allocation** | 105  (1 RCT)  [18] | RD= –0.06 (–0.17 to 0.05) | ⊕⊖⊖⊖  Very low | Imprecision^1^ |  |
| **Completed suicides** | 0  (0 RCT) | - | - | - | Outcome not reported in study |
| **Suicidal ideation** | 0  (0 RCT) | - | - | - | Outcome not reported in study |
| **Depression** | 0  (0 RCT) | - | - | - | Outcome not reported in study |
| **Anxiety** | 0  (0 RCT) | - | - | - | Outcome not reported in study |
| **General function** | 0  (0 RCT) | - | - | - | Outcome not reported in study |

**CI** = Confidence Interval; **GRADE** = The Grading of Recommendations Assessment, Development and Evaluation; **RCT** = Randomized controlled trial; **RD** = Risk Difference; **TAU** =Treatment As Usual

^1^ Downrated –3 because of imprecision: non-significant results, few participants an only one study

### Group therapy versus TAU

#### Metaanalyses

**Figure S25** Repetition of self-harm* at 6 months post-allocation**.


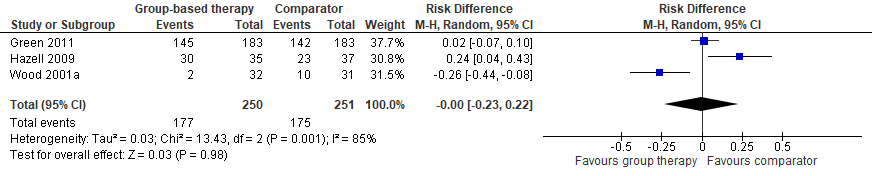


* All studies reported interviewed-assessed self-harm during last 6 months. No study reported SA or NSSI

** Post-intervention analysis was not performed since it was not clear in all studies when the interventions stopped

**NSSI** = Non-Suicidal Self-Injury; **SA** = Suicide Attempts

**Figure S26** Repetition of self-harm at 12 months post-allocation.


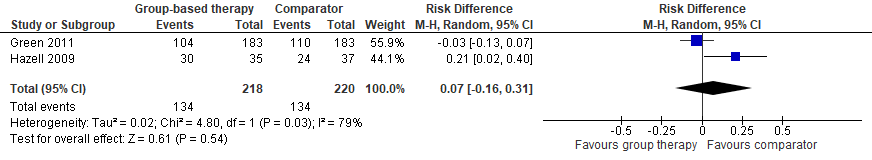


**Figure S27** Suicidal ideation scores (SIQ) at 6 months post-allocation.


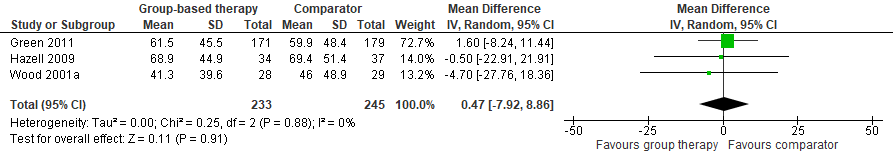


**SIQ** = Suicidal Ideation Questionnaire (range 0–180)

**Figure S28** Suicidal ideation scores (SIQ) at 12 months post-allocation.


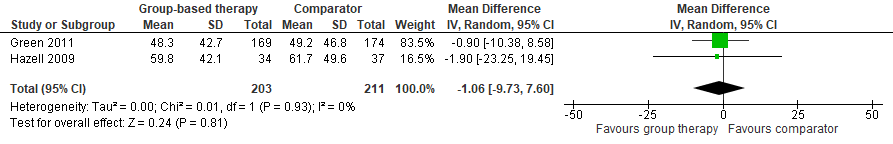


**SIQ** = Suicidal Ideation Questionnaire (range 0–180)

**Figure S29** Depression scores (MFQ) at 6 months.


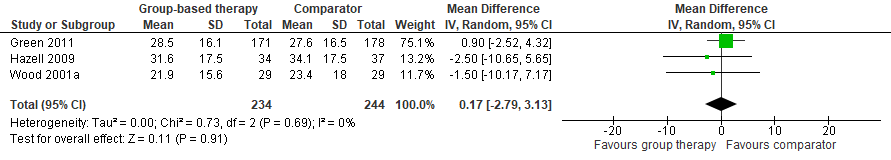


**MFQ** = Mood and Feelings Questionnaire (range 0–26)

**Figure S30** Depression scores (MFQ) at 12 months.


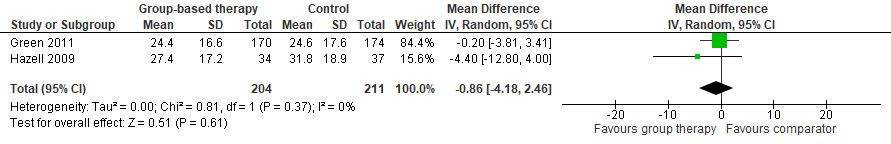


**MFQ** = Mood and Feelings Questionnaire (range 0–26)

**Figure S31** General functioning scores (HoNOSCA) at 6 months.


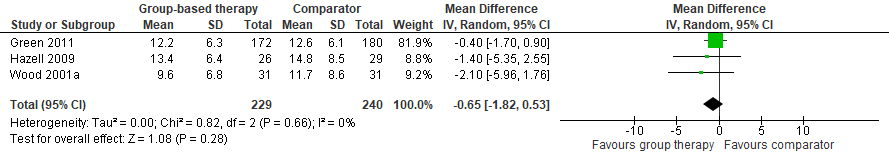


**HoNOSCA** = Health of the Nation Outcome Scales (range 0–52)

**Figure S32** General functioning scores (HoNOSCA) at 12 months.


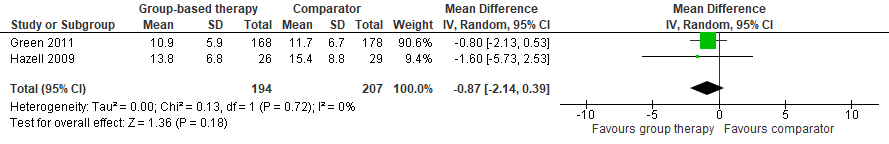


**HoNOSCA** = Health of the Nation Outcome Scales (range 0–52)

#### Table S10. Summary of findings for group therapy versus TAU.

| Utfallsmått | Antal deltagare  (Antal studier, Studiedesign)  Reference | Effekt  (95% KI) | Resultatets tillförlitlighet | Avdrag | | Kommentar | |  |
| --- | --- | --- | --- | --- | --- | --- | --- | --- |
| **Number of participants with self-harm at 6 months post-allocation** | 501  (3, RCT)  [19-21] | RD= –0.00 (–0.23 to 0.22) | ⊕⊖⊖⊖  Very low | | Imprecision,  Inconsistency^1^ | |  | |
| **Number of participants with self-harm at 12 months post-allocation** | 438  (2 RCT) | RD=0.07 (–0.16 to 0.31) | ⊕⊖⊖⊖  Very low | Imprecision,  Inconsistency^1^ | |  | |  |
| **Suicide attempts** | 0  (0 RCT) | - | − | - | | Outcome not reported in any study | |  |
| **Completed suicides** | 478  (3 RCT) | Group therapy: 0  TAU: 0 | ⊕⊖⊖⊖  Very low | Imprecision^2^ | | Metaanalysis not performed because of 0 events | |  |
| **Suicidal ideation scores (SIQ) at 6 months post-allocation** | 478  (3 RCT)  [19-21] | MD=0.47 (–7.92 to 8.86) | ⊕⊕⊕⊖  Moderate | Imprecision and risk of bias^3^ | | Group therapy probably results in little to no difference | |  |
| **Suicidal ideation scores (SIQ) at 12 months post-allocation** | 414  (2 RCT)  [19, 20] | MD=–1.06 (–9.73 to 7.60) | ⊕⊕⊕⊖  Moderate | Imprecision and risk of bias^3^ | | Group therapy probably results in little to no difference | |  |
| **Depression scores (MFQ) at 6 months post-allocation** | 478  (3 RCT)  [19-21] | MD=0.17 (–2.79 to 3.13) | ⊕⊖⊖⊖  Very low | Imprecision^4^ | |  | |  |
| **Depression scores (MFQ) at 12 months post-allocation** | 415  (2 RCT)  [19, 20] | MD=0.86 (–4.18 to 2.46) | ⊕⊖⊖⊖  Very low | Imprecision^4^ | |  | |  |
| **Anxiety** | 0  (0 RCT) | - | - | - | | Outcome not reported in any study | |  |
| **General function scores (HoNOSCA) at 6 months post-allocation** | 469  (3 RCT)  [19-21] | MD= –0.65 (–1.82 to 0.53) | ⊕⊖⊖⊖  Very low | Imprecision^4^ | |  | |  |
| **General function scores (HoNOSCA) at 12 months post-allocation** | 401  (2 RCT)  [19, 20] | MD= –0.87 (–2.14 to 0.39) | ⊕⊖⊖⊖  Very low | Imprecision^4^ | |  | |  |

**CI** = Confidence Interval; **GRADE** = The Grading of Recommendations Assessment, Development and Evaluation; **HoNOSCA** = Health of the Nation Outcome Scales (range 0–52); **MD** = Mean Difference; **MFQ** = Mood and Feelings Questionnaire (range 0–26); **RCT** = Randomized controlled trial; **RD** = Risk Difference; **SIQ** = Suicidal Ideation Questionnaire (range 0–180); **TAU** =Treatment As Usual

^1^ Downrated –2 because of imprecision: CI includes both considerable benefit and harm, and -1 because of inconsistency: the results of the three studies are very different

^2^ Downrated –3 because of imprecision: very few events Downrated –2 because of imprecision: CI includes both important benefit and harm, and -1 because of inconsistency: the results of the three studies are very different

^3^ Downrated -1 because of precision: few participants; and risk of bias: some concerns regarding randomization and reporting

^4^ Downrated –3 because of imprecision: non-significant results and few participants

### Family therapy

#### Table S11. Summary of findings for systemic family therapy versus TAU.

| Outcome | Number of participants  (Number of studies)  References | Effect  (95% CI) | Certainty of the evidence (GRADE) | Downrating (GRADE) | Comment |
| --- | --- | --- | --- | --- | --- |
| **Number of participants with self-harm, at post-treatment** | 0  (0 RCT) | - | - | - | Outcome only reported at later follow-up |
| **Number of participants with self-harm, at follow-up** | 832  (1 RCT)  [22-24] | At 12 months post-allocation:  RD=0.04 (–0.02 to 0.10)  At 3 years post-allocation:  RD=0.01 (–0.06 to 0.07) | ⊕⊕⊖⊖  Low | Imprecision^1^ | Evidence suggests family therapy results in little to no difference |
| **Suicide attempts** | 0  (0 RCT) | - | - | - | Outcome not reported in study |
| **Completed suicides, at 3 years follow-up** | 832  (1 RCT)  [24] | FT: 1  TAU: 0 | ⊕⊖⊖⊖  Very low | Imprecision^2^ | Meta-analysis not performed because of 0 events |
| **Number of participants with suicidal ideation (BSSI screening), at 12 months post-allocation** | 832  (1 RCT)  [22, 23] | OR=0.64 (0.44 to 0.94) | ⊕⊕⊕⊖  Moderate | Risk of bias and imprecision^3^ | Family therapy probably reduces outcome. Data for calculating RD was not reported in study |
| **Number of participants with suicidal ideation (BSSI screening), at 18 months post-allocation** | 832  (1 RCT)  [24] | OR=0.76 (0.49 to 1.16) | ⊕⊖⊖⊖  Very low | Imprecision^4^ | Data for calculating RD was not reported in study |
| **Depression** | 832  (1 RCT)  [22-24] | At 12 months post-allocation: MD= –0.6 (–3.1 to 1.9)  At 18 months post-allocation: MD= –1.0 (–3.5 to 1.5) | ⊕⊖⊖⊖  Very low | Imprecision^4^ | Multiple imputation of missing data was used by study authors |
| **Anxiety** | 0  (0 RCT) | - | - | - | Outcome not reported in study |
| **General function** | 0  (0 RCT) | - | - | - | Outcome not reported in study |

**BSSI** = Beck Scale for Suicide Ideation (range 0–38); **CI** = Confidence Interval; **GRADE** = The Grading of Recommendations Assessment, Development and Evaluation; **MD** = Mean Difference; **RCT** = Randomized controlled trial; **RD** = Risk Difference; **TAU** =Treatment As Usual

^1^ Downrated –2 because of precision: few participants (only one study)

^2^ Downrated –3 because of imprecision: very few events and few particiapants (only one study)

^3^ Downrated –1 because of risk of bias: high attrition rate; and precision: few participants (only one study)

^4^ Downrated –3 because of non-significant results and few participants (only one study)

#### Table S12. Summary of findings for home-based family therapy versus TAU.

| Outcome | Number of participants  (Number of studies)  References | Absolute effect  (95% CI) | Certainty of the evidence (GRADE) | Downrating (GRADE) | Comment |
| --- | --- | --- | --- | --- | --- |
| **Number of participants with self-harm, at 6 months post-allocation** | 162  (1 RCT)  [25] | At 6 months post-allocation:  RD= –0.01 (–0.12 to 0.09) | ⊕⊖⊖⊖  Very low | Imprecision^1^ |  |
| **Suicide attempts** | 0  (0 RCT) | - | - | - | Outcome not reported in study |
| **Completed suicides** | 162  (1 RCT) | FT: 1  TAU: 0 | ⊕⊖⊖⊖  Very low | Imprecision^2^ | Metaanalysis not performed because of very few events |
| **Suicidal ideation (SIQ)** | 154  (1 RCT) | At 2 months post-allocation: MD= –3.40 (–19.18 to 12.38)  At 6 months post-allocation: MD= –5.10 (–17.37 to 7.17) | ⊕⊖⊖⊖  Very low | Imprecision^1^ |  |
| **Depression, diagnosis of major depression** | 162  (1 RCT) | At 2 months post-allocation: RD=0.05 (–0.09 to 0.18)  At 6 months post-allocation: RD= –0.08 (–0.20 to 0.04) | ⊕⊖⊖⊖  Very low | Imprecision^1^ | Clinical diagnosis of depression according to DSM-IV |
| **Anxiety** | 0  (0 RCT) | - | - | - | Outcome not reported in study |
| **General function** | 0  (0 RCT) | - | - | - | Outcome not reported in study |

**CI** = Confidence Interval; **GRADE** = The Grading of Recommendations Assessment, Development and Evaluation; **MD** = Mean Difference; **RCT** = Randomized controlled trial; **RD** = Risk Difference; **SIQ** = Suicidal Ideation Questionnaire (range 0–180); **TAU** =Treatment As Usual

^1^ Downrated –3 because of non-significant results and few participants

^2^ Downrated –3 because of imprecision: very few events

## **References**

1. Asarnow JR, Hughes JL, Babeva KN, Sugar CA. Cognitive-Behavioral Family Treatment for Suicide Attempt Prevention: A Randomized Controlled Trial. J Am Acad Child Adolesc Psychiatry. 2017;56(6):506-14. Available from: <https://doi.org/10.1016/j.jaac.2017.03.015>.

2. Donaldson D, Spirito A, Esposito-Smythers C. Treatment for adolescents following a suicide attempt: results of a pilot trial. J Am Acad Child Adolesc Psychiatry. 2005;44(2):113-20. Available from: <https://doi.org/10.1097/00004583-200502000-00003>.

3. Duarte-Velez Y, Jimenez-Colon G, Jones RN, Spirito A. Socio-Cognitive Behavioral Therapy for Latinx Adolescent with Suicidal Behaviors: A Pilot Randomized Trial. Child Psychiatry Hum Dev. 2022. Available from: <https://doi.org/10.1007/s10578-022-01439-z>.

4. Esposito-Smythers C, Wolff JC, Liu RT, Hunt JI, Adams L, Kim K, et al. Family-focused cognitive behavioral treatment for depressed adolescents in suicidal crisis with co-occurring risk factors: a randomized trial. J Child Psychol Psychiatry. 2019;60(10):1133-41. Available from: <https://doi.org/10.1111/jcpp.13095>.

5. Kaess M, Edinger A, Fischer-Waldschmidt G, Parzer P, Brunner R, Resch F. Effectiveness of a brief psychotherapeutic intervention compared with treatment as usual for adolescent nonsuicidal self-injury: a single-centre, randomised controlled trial. Eur Child Adolesc Psychiatry. 2020;29(6):881-91. Available from: <https://doi.org/10.1007/s00787-019-01399-1>.

6. Rockstroh F, Edinger A, Josi J, Fischer-Waldschmidt G, Brunner R, Resch F, Kaess M. Brief Psychotherapeutic Intervention Compared with Treatment as Usual for Adolescents with Nonsuicidal Self-Injury: Outcomes over a 2-4-Year Follow-Up. Psychother Psychosom. 2023;92(4):243-54. Available from: <https://doi.org/10.1159/000531092>.

7. McCauley E, Berk MS, Asarnow JR, Adrian M, Cohen J, Korslund K, et al. Efficacy of dialectical behavior therapy for adolescents at high risk for suicide a randomized clinical trial. JAMA Psychiatry. 2018;75(8):777-85. Available from: <https://doi.org/10.1001/jamapsychiatry.2018.1109>.

8. Mehlum L, Tormoen AJ, Ramberg M, Haga E, Diep LM, Laberg S, et al. Dialectical behavior therapy for adolescents with repeated suicidal and self-harming behavior: a randomized trial. J Am Acad Child Adolesc Psychiatry. 2014;53(10):1082-91. Available from: <https://doi.org/10.1016/j.jaac.2014.07.003>.

9. Santamarina-Perez P, Mendez I, Singh MK, Berk M, Picado M, Font E, et al. Adapted Dialectical Behavior Therapy for Adolescents with a High Risk of Suicide in a Community Clinic: A Pragmatic Randomized Controlled Trial. Suicide Life Threat Behav. 2020;50(3):652-67. Available from: <https://doi.org/10.1111/sltb.12612>.

10. Mehlum L, Ramberg M, Tormoen AJ, Haga E, Diep LM, Stanley BH, et al. Dialectical Behavior Therapy Compared With Enhanced Usual Care for Adolescents With Repeated Suicidal and Self-Harming Behavior: Outcomes Over a One-Year Follow-Up. J Am Acad Child Adolesc Psychiatry. 2016;55(4):295-300. Available from: <https://doi.org/10.1016/j.jaac.2016.01.005>.

11. Mehlum L, Ramleth RK, Tormoen AJ, Haga E, Diep LM, Stanley BH, et al. Long term effectiveness of dialectical behavior therapy versus enhanced usual care for adolescents with self-harming and suicidal behavior. J Child Psychol Psychiatry. 2019;60(10):1112-22. Available from: <https://doi.org/10.1111/jcpp.13077>.

12. Bjureberg J, Ojala O, Hesser H, Habel H, Sahlin H, Gratz KL, et al. Effect of Internet-Delivered Emotion Regulation Individual Therapy for Adolescents With Nonsuicidal Self-Injury Disorder: A Randomized Clinical Trial. JAMA Netw Open. 2023;6(7):e2322069. Available from: <https://doi.org/10.1001/jamanetworkopen.2023.22069>.

13. Morthorst B, Olsen MH, Jakobsen JC, Lindschou J, Gluud C, Heinrichsen M, et al. Internet based intervention (Emotion Regulation Individual Therapy for Adolescents) as add-on to treatment as usual versus treatment as usual for non-suicidal self-injury in adolescent outpatients: The TEENS randomised feasibility trial. JCPP Adv. 2022;2(4):e12115. Available from: <https://doi.org/10.1002/jcv2.12115>.

14. Griffiths H, Duffy F, Duffy L, Brown S, Hockaday H, Eliasson E, et al. Efficacy of Mentalization-based group therapy for adolescents: the results of a pilot randomised controlled trial. BMC Psychiatry. 2019;19(1):167. Available from: <https://doi.org/10.1186/s12888-019-2158-8>.

15. Rossouw TI, Fonagy P. Mentalization-based treatment for self-harm in adolescents: a randomized controlled trial. J Am Acad Child Adolesc Psychiatry. 2012;51(12):1304-13 e3. Available from: <https://doi.org/10.1016/j.jaac.2012.09.018>.

16. Kennard BD, Goldstein T, Foxwell AA, McMakin DL, Wolfe K, Biernesser C, et al. As Safe as Possible (ASAP): A Brief App-Supported Inpatient Intervention to Prevent Postdischarge Suicidal Behavior in Hospitalized, Suicidal Adolescents. Am J Psychiatry. 2018;175(9):864-72. Available from: <https://doi.org/10.1176/appi.ajp.2018.17101151>.

17. Ougrin D, Zundel T, Ng A, Banarsee R, Bottle A, Taylor E. Trial of Therapeutic Assessment in London: randomised controlled trial of Therapeutic Assessment versus standard psychosocial assessment in adolescents presenting with self-harm. Arch Dis Child. 2011;96(2):148-53. Available from: <https://doi.org/10.1136/adc.2010.188755>.

18. Cotgrove A, Zirinsky L, Black D, Weston D. Secondary prevention of attempted suicide in adolescence. J Adolesc. 1995;18(5):569-77. Available from: <https://doi.org/10.1006/jado.1995.1039>.

19. Green JM, Wood AJ, Kerfoot MJ, Trainor G, Roberts C, Rothwell J, et al. Group therapy for adolescents with repeated self harm: randomised controlled trial with economic evaluation. BMJ. 2011;342:d682. Available from: <https://doi.org/10.1136/bmj.d682>.

20. Hazell PL, Martin G, McGill K, Kay T, Wood A, Trainor G, Harrington R. Group therapy for repeated deliberate self-harm in adolescents: failure of replication of a randomized trial. J Am Acad Child Adolesc Psychiatry. 2009;48(6):662-70. Available from: <https://doi.org/10.1097/CHI.0b013e3181aOacec>.

21. Wood A, Trainor G, Rothwell J, Moore A, Harrington R. Randomized trial of group therapy for repeated deliberate self-harm in adolescents. J Am Acad Child Adolesc Psychiatry. 2001;40(11):1246-53. Available from: <https://doi.org/10.1097/00004583-200111000-00003>.

22. Cottrell DJ, Wright-Hughes A, Collinson M, Boston P, Eisler I, Fortune S, et al. Effectiveness of systemic family therapy versus treatment as usual for young people after self-harm: a pragmatic, phase 3, multicentre, randomised controlled trial. Lancet Psychiatry. 2018;5(3):203-16. Available from: <https://doi.org/10.1016/S2215-0366(18)30058-0>.

23. Cottrell DJ, Wright-Hughes A, Collinson M, Boston P, Eisler I, Fortune S, et al. A pragmatic randomised controlled trial and economic evaluation of family therapy versus treatment as usual for young people seen after second or subsequent episodes of self-harm: the Self-Harm Intervention - Family Therapy (SHIFT) trial. Health Technol Assess. 2018;22(12):1-222. Available from: <https://doi.org/10.3310/hta22120>.

24. Cottrell DJ, Wright-Hughes A, Eisler I, Fortune S, Green J, House AO, et al. Longer-term effectiveness of systemic family therapy compared with treatment as usual for young people after self-harm: An extended follow up of pragmatic randomised controlled trial. EClinicalMedicine. 2020;18:100246. Available from: <https://doi.org/10.1016/j.eclinm.2019.100246>.

25. Harrington R, Kerfoot M, Dyer E, McNiven F, Gill J, Harrington V, et al. Randomized Trial of a Home-Based Family Intervention for Children Who Have Deliberately Poisoned Themselves. J Am Acad Child Adolesc Psychiatry. 1998;37(5):512-8. Available from: <https://doi.org/10.1016/s0890-8567(14)60001-0>.
